# Psychological Stress Disrupts Goblet Cell Function and Intestinal Barrier via 5-HT/HTR7-Mediated NLRP6 and Autophagy Suppression

**DOI:** 10.3390/biology15201870

**Published:** 2026-10-16

**Authors:** Shuo Wang, Hongwei Zhu, Youzhi Li, Jizhen Zou, Ping Mu, Xin Yu, Yang Liu, Jianlong Zhang, Linlin Jiang, Jiayu Yu, Xingxiao Zhang

**Affiliations:** 1 School of Life Sciences, Ludong University, Yantai 264025, China; 2 Collaborative Innovation Center for the Pet Infectious Diseases and Public Health in the Middle and Lower Stream Regions of the Yellow River, Yantai 264025, China; 3 Shandong Engineering Research Center for Aquaculture Environment Control, Yantai 264025, China; 4 Shandong Provincial Key Laboratory of Livestock Product Quality and Safety Supervision & New Veterinary Drug Creation, Ji’nan 250022, China; 5 Wendeng District Animal Husbandry and Veterinary Undertaking Development Center of Weihai City, Weihai 264400, China

**Keywords:** autophagy, 5-HT/HTR7, intestinal mucosal barrier, NLRP6, PPAR-γ

## Abstract

Psychological stress is closely associated with intestinal barrier dysfunction, yet the underlying mechanisms remain incompletely understood. Here, using a 14-day restraint stress mouse model and an HT-29 cell model, we demonstrate that stress-induced elevation of serotonin (5-HT) disrupts intestinal mucosal barrier function through the 5-HT/HTR7/PPAR-γ/NLRP6/autophagy axis. Restraint stress significantly increased 5-HT levels in serum and intestinal tissues, accompanied by reduced goblet cell numbers, impaired mucin 2 secretion, and increased intestinal permeability. Mechanistically, 5-HT acts via HTR7 to downregulate PPAR-γ, which transcriptionally controls NLRP6, thereby suppressing NLRP6 inflammasome activity and autophagy. Pharmacological inhibition of 5-HT synthesis with PCPA or activation of PPAR-γ with rosiglitazone reversed these pathological phenotypes in vivo, and the 5-HT/HTR7/PPAR-γ axis was further validated in vitro. These findings establish a novel downstream mechanism linking psychological stress to NLRP6 inflammasome dysfunction, impaired autophagy, defective goblet cell function, and intestinal mucosal barrier damage.

## Simple Summary

Psychological stress can harm our digestive system, but how this happens is not fully understood. This study aimed to find out how stress damages the protective lining of the gut. Researchers used mice that were placed under stress for 14 days, as well as cells grown in the lab. They found that stress increases the amount of a chemical called serotonin in the blood and gut. High serotonin then triggers a chain reaction that blocks certain protective proteins, weakening the gut’s defense system. Specifically, it reduces the mucus-producing cells and makes the gut more leaky. Importantly, when the researchers blocked serotonin or activated a specific protective protein, the damage was reversed. This study reveals a complete pathway explaining how stress causes gut injury. These findings are valuable because they could help scientists develop new treatments to protect people’s digestive health during stressful times.

## 1. Introduction

The intestinal response to psychological stress is characterized by altered intestinal peristalsis, elevated visceral sensitivity, and increased intestinal permeability [1]. Chronic psychosocial stress increases the risk of intestinal diseases, including irritable bowel syndrome (IBS) [1]. Accumulating evidence suggests that serotonin (5-hydroxytryptamine, 5-HT) signaling is altered in IBS, reflected by changes in enterochromaffin (EC) cell numbers, 5-HT level, tryptophan hydroxylase 1 (TPH1) expression, serum 5-HT level, and 5-HT-selective reuptake transporter (SERT) expression [2]. However, the intestine is the major source of biologically active 5-HT, with most 5-HT being synthesized by a subpopulation of epithelial cells called EC cells [3]. EC cells are dispersed in the epithelial cells of the gastrointestinal mucosal layer. In response to various mechanical and chemical stimuli, they release 5-HT into the intestinal lumen at the top and bottom. However, how changes in gut 5-HT signaling affect the disease development mechanism of IBS is still unclear.

Intestinal permeability is increased in patients with IBS [4], resulting in a “leaky gut.” This compromised barrier facilitates the entry of pathogens and proinflammatory substances. The tight epithelium forms a physical barrier that is further protected by the mucus layer, which provides protective functions at different sites [5]. Goblet cells provide the protective mucus barrier that covers the intestine, serving as a crucial antimicrobial protective mechanism. The intestinal mucus layer consists of two layers and plays a crucial role in maintaining intestinal homeostasis. It protects the intestinal epithelium from dehydration, physical wear and tear, as well as symbiotic and invasive microorganisms [6,7]. The dense internal mucus layer is devoid of microbial communities and serves as a physical barrier to minimize microbial translocation and prevent excessive immune activation [6]. However, the regulatory pathways by which goblet cells induce mucus secretion remain poorly understood.

Nucleotide-binding oligomerization domain (NOD)-like receptor family pyrin domain containing 6 (NLRP6) is a new member of the NOD-like receptor (NLR) family. Upon activation, NLRP6 recruits adaptor proteins known as apoptosis-associated speckle-like proteins (ASC) for the formation of functional inflammasomes, which subsequently leads to proteolysis and activation of caspase-1/11 and release of the proinflammatory cytokines interleukin (IL)-1β and IL-18 [8,9]. NLRP6 is highly expressed in mouse intestinal epithelial cells and is critical for protecting against the development of enteritis by regulating the stability of the intestinal mucosal barrier [10,11,12]. In a mouse model of stress-induced enteritis, water avoidance stress (WAS) inhibited the expression of NLRP6 and disrupted the NLRP6 inflammasome signaling pathway, resulting in pathological changes in the small intestine [13]. Furthermore, studies have shown that NLPR6^−/−^ mice exhibited significant goblet cell hyperplasia. However, the secretion of mucus granules by goblet cells was inhibited, and the continuous adherent inner mucosal layer was absent [14].

This study employed a murine restraint stress model to elucidate the role of 5-HT in stress-induced intestinal pathology. The results demonstrated that restraint stress elevated intestinal 5-HT levels, downregulated tight junction (Occludin, Claudin-3) proteins, and impaired mucin 2 (MUC2) secretion, collectively disrupting the intestinal mucosal barrier. Concurrently, the expression of peroxisome proliferator-activated receptor gamma (PPAR γ), NLRP6 inflammasome components, and autophagy-related proteins was markedly suppressed in the intestinal epithelium. Notably, pharmacological blockade of 5-HT synthesis in restraint-stressed mice significantly restored the expression of PPAR-γ, NLRP6 inflammasome, and autophagic markers, thereby ameliorating mucosal barrier injury. In vitro mechanistic studies further demonstrated that 5-HT acts through the 5-hydroxytryptamine receptor 7 (HTR7) to suppress PPAR-γ expression, which, in turn, attenuates NLRP6 inflammasome activation and autophagy, culminating in reduced mucin secretion by colorectal adenocarcinoma HT-29 cells. Collectively, these findings identified a previously unrecognized 5-HT/HTR7/PPAR-γ/NLRP6/autophagy axis that mediates stress-induced intestinal mucosal barrier dysfunction.

## 2. Materials and Methods

### 2.1. Ethics Statement

This study received approval from the Institutional Animal Care and Use Committee at Ludong University on 22 June 2024 (Authorization Permit Number: LDU-IRB20240622). The animal experiments were conducted in accordance with the guidelines outlined in the People’s Republic of China National Standard GB/T 35892–2018 [15] for the ethical review of laboratory animal welfare.

### 2.2. Animal Treatments

All experimental animals were female C57BL/6J mice aged 6–7 weeks, purchased from Vital River Laboratory Animal Technology Co., Ltd. (Beijing, China). Mice were housed under specific pathogen-free (SPF) conditions with a 14 h light/dark cycle and had ad libitum access to standard rodent feed and filtered water during the entire experimental period.

A total of 120 mice were used in this study, including mice for drug concentration gradient screening experiments and formal animal experiments. The sample size of each group (n = 6 or n = 10) was determined based on relevant published studies on stress-induced intestinal injury and statistical power analysis. This setting can effectively detect biological differences among groups and comply with the 3R principles of laboratory animal welfare to reduce unnecessary animal use. All mice were numbered before grouping, and group allocation was performed using a random number table by an independent researcher. Researchers who performed animal modeling, drug administration, sample collection and data analysis were blinded to group information to avoid artificial experimental bias.

After a one-week adaptive acclimation period, all mice were randomly divided into two experimental parts: drug concentration gradient screening experiment and formal animal experiment.

(1). Drug concentration gradient screening experiment

A total of 60 mice were used to explore the concentration gradients of para-chlorophenylalanine (PCPA) and rosiglitazone. PCPA is a selective inhibitor of tryptophan hydroxylase 1 (TPH1), the rate-limiting enzyme for peripheral serotonin (5-HT) synthesis, and is commonly used to deplete endogenous 5-HT. Rosiglitazone is a selective PPARγ agonist used to pharmacologically activate PPARγ signaling. The grouping and dosages were as follows:

Control group (C), n = 6;

Restraint stress group (S), n = 6;

Restraint stress + low-dose PCPA (S + P-L, 50 mg/kg), n = 6;

Restraint stress + medium-dose PCPA (S + P-M, 100 mg/kg), n = 6;

Restraint stress + high-dose PCPA (S + P-H, 150 mg/kg), n = 6;

Restraint stress + low-dose rosiglitazone (S + R-L, 10 mg/kg), n = 6;

Restraint stress + medium-dose rosiglitazone (S + R-M, 20 mg/kg), n = 6;

Restraint stress + high-dose rosiglitazone (S + R-H, 30 mg/kg), n = 6;

Single PCPA treatment group (P-H, 150 mg/kg), n = 6;

Single rosiglitazone treatment group (R-H, 30 mg/kg), n = 6.

(2). Formal animal experiment

A total of 60 mice were used for formal validation experiments based on the optimal drug concentrations screened above.

Experiment 1

Twenty mice were divided into two groups (n = 10 per group):

Control group (C)

Restraint stress group (S)

Experiment 2

Forty mice were divided into four groups (n = 10 per group):

Control group (C)

Restraint stress group (S)

Restraint stress + PCPA group (S + P, 150 mg/kg)

Restraint stress + rosiglitazone group (S + R, 30 mg/kg)

(3). Unified modeling and drug administration protocol

Chronic restraint stress was performed uniformly in all stress-related groups. Each mouse was placed individually in a ventilated transparent 50 mL plastic centrifuge tube for 6 h per day (10:00 a.m. to 4:00 p.m.) for 14 consecutive days.

Two hours before each restraint treatment, PCPA (C6506, Sigma-Aldrich, St. Louis, MO, USA) at corresponding doses was administered via intraperitoneal injection, and rosiglitazone (R2408, Sigma-Aldrich, St. Louis, MO, USA) at corresponding doses was given by oral gavage. Mice in the control group received an equal volume of vehicle solution composed of 10% DMSO, 40% PEG300, 5% Tween-80 and 45% sterile saline.

All drugs were first prepared as 10-fold stock solutions using DMSO, and then diluted 10-fold with the above vehicle. A volume of 100 μL working solution was administered to each mouse via intraperitoneal injection or oral gavage according to experimental requirements. The reagents used included DMSO (D8418, Sigma-Aldrich, St. Louis, MO, USA), PEG300 (HY-Y0873, MCE, Monmouth Junction, NJ, USA), Tween-80 (HY-Y0873, MCE, Monmouth Junction, NJ, USA) and sterile saline.

### 2.3. Intestinal Permeability

Intestinal barrier permeability in vivo was assessed using the FITC-dextran (FD-4, 4 kDa) assay to evaluate intestinal barrier integrity. After an overnight period of food and water deprivation, mice were orally administered FITC-labeled dextran (FD-4, Cat. No. 46944; Sigma-Aldrich) at a dose of 60 mg per 100 g body weight. Serum samples were collected 5 h after FD-4 gavage, and the fluorescence intensity of each specimen was measured at an excitation wavelength of 492 nm and an emission wavelength of 525 nm. A standard curve was established by serially diluting FITC-dextran in phosphate-buffered saline (PBS). The serum concentration of FITC-dextran was then quantified based on the prepared standard curve.

### 2.4. Histological Analysis

Following washing with PBS, intestinal samples were fixed with 4% paraformaldehyde and prepared for paraffin embedding. The tissues were cut into 5 μm sections, which were then dewaxed and rehydrated. Subsequently, the sections were stained with Alcian Blue Periodic acid Schiff (AB PAS) staining kit (Cat. G1285, Solarbio, Beijing, China) to identify goblet cells. Goblet cells were counted along a 100 μm intestinal segment; at least 30 random fields were captured from six sections per sample under an Olympus BX51 microscope (Olympus BX51 microscope, Tokyo, Japan) at 400× magnification. Immunohistochemical staining was performed to detect MUC2 (Cat. ab272692, Abcam, Cambridge, UK, 1:1000 dilution) and Claudin 3 (Cat. 16456 1 AP, Proteintech, Rosemont, IL, USA, 1:800 dilution) in paraffin-embedded intestinal sections. Sections were incubated overnight at 4 °C with rabbit anti-MUC2 primary antibody. After washing with PBS (pH 7.4), the sections were incubated with biotinylated goat anti-rabbit IgG secondary antibody (Cat. ab64256, Abcam, 1:1000 dilution) for 2 h, followed by 1:300 diluted HRP streptavidin (Cat. ab7403, Abcam) for another 2 h. Immunoreactive signals were visualized using a diaminobenzidine (DAB) kit (Cat. ZLI 9017, Zhongshan Jinqiao Biotechnology Co., Ltd. ZSBIO, Beijing, China), and nuclei were counterstained with hematoxylin for 5 min. The mean integrated optical density (IOD) of positively stained areas was quantified using ImageJ software (version 1.54f; National Institutes of Health, Bethesda, MD, USA). For immunofluorescent staining of PPAR γ (Cat. ab272718, Abcam, 1:1000 dilution), the sections were incubated overnight at 4 °C with rabbit anti-PPAR γ primary antibody. After rinsing with PBS on a decolorizing shaker, the sections were incubated with Alexa Fluor 594-conjugated goat anti-rabbit secondary antibody (Cat. ab150080, Abcam, 1:1000 dilution) at room temperature for 60 min. Fluorescent images were captured using a Nikon Eclipse TE 2000S inverted microscope (Nikon Instruments, Inc., Melville, NY, USA), and the number of positively stained puncta was analyzed with ImageJ (ImageJ, Bethesda, MD, USA) software.

### 2.5. RNA Isolation and RT–qPCR Analysis

Intestinal tissues were collected from experimental mice and homogenized in TRIzol reagent, and total RNA was extracted in accordance with the manufacturer’s protocol. One microgram of purified RNA was reverse-transcribed into complementary DNA (cDNA) using HiScript II Select qRT SuperMix (Cat. R312-02, Vazyme, Nanjing, China). Real-time quantitative PCR (RT–qPCR) was carried out using gene-specific primers and SYBR Green qPCR Master Mix (Cat. Q141-0, Vazyme). Fluorescence signals were detected using an Applied Biosystems StepOnePlus Real-Time PCR System (Applied Biosystems StepOnePlus Real-Time PCR System, Waltham, MA, USA). The real-time qPCR thermal cycling program was set as follows: initial denaturation at 95 °C for 3 min, followed by 40 cycles of denaturation at 95 °C for 10 s and combined annealing-extension at 60 °C for 30 s. A melting-curve analysis (95 °C for 15 s, 60 °C for 60 s, continuous heating to 95 °C) was performed at the end of amplification to verify the specificity of PCR products. The primer sequences employed in this study are listed in Table S1.

### 2.6. Western Blotting

Total proteins were extracted from intestinal tissues and cell lines using RIPA lysis buffer (Cat. R0010, Solarbio, Beijing, China) supplemented with protease and phosphatase inhibitors (Cat. P1045, Beyotime Biotechnology, Shanghai, China) and incubated for 30 min. Protein concentrations were determined using a BCA protein quantification kit (Cat. CW0014S, Kangwei Century Biotechnology Co., Ltd., CWBIO, Taizhou, China). Equal amounts of protein were separated by 10–15% SDS-PAGE and then electrotransferred onto polyvinylidene fluoride (PVDF) membranes. The membranes were blocked with 5% skimmed milk in Tris-buffered saline containing Tween 20 (TBST, Tris-buffered saline containing 0.1% (*v*/*v*) Tween-20) at room temperature for 1.5 h. Subsequently, the membranes were incubated overnight at 4 °C with the corresponding primary antibodies. After washing, the membranes were incubated with horseradish peroxidase (HRP)-conjugated secondary antibodies for 2 h at room temperature. Protein bands were visualized using an ECL Western blot detection kit (Cat. CW0049; CoWin Biotech Co., Inc., Beijing, China). The intensity of the target bands was scanned and quantified using ImageJ software (National Institutes of Health, Bethesda, MD, USA). The intensity of each target protein band was normalized to β-actin (loading control) from the same blot. All Western blot experiments were repeated with ≥3 independent biological replicates. Full-length uncropped Western blot images are provided in Supplementary Figure S3. Detailed information for all Western blot antibodies, including target names, vendors, catalog numbers, RRIDs, and working dilutions, is summarized in Supplementary Table S2.

### 2.7. Measurement of Serotonin Concentration in Intestinal Tissues

Serotonin levels in intestinal tissues were quantified using a competitive enzyme-linked immunosorbent assay (ELISA) kit. The detection range of the assay was 0–80 nmol/mL. All experimental procedures were conducted strictly in accordance with the manufacturer’s instructions.

### 2.8. Cell Treatment

Human colorectal adenocarcinoma HT-29 cells (ATCC HTB-38, RRID: CVCL_0320) were maintained in Dulbecco’s Modified Eagle’s Medium (DMEM, Vivacell, Shanghai, China) supplemented with 10% fetal bovine serum (FBS) and penicillin/streptomycin (100 ng/mL), and cultured at 37 °C in a humidified atmosphere containing 5% CO_2_. For experimental treatments, cells were seeded into 48-well plates at a density of 1 × 10^4^ cells/mL and 6-well plates at 1 × 10^6^ cells/mL, respectively. The cells were validated by short tandem repeats and tested negative for mycoplasma. After overnight incubation to allow cell adhesion, the cells were washed twice with PBS and incubated in serum-free medium. In the 5 HT and SB 269970 intervention groups, cells were pre-treated with 20 μM SB 269970 (a 5 HT7 receptor antagonist) for 2 h, followed by stimulation with 10 μM 5 HT for 24 h. Separately, cells were treated with 10 μM rosiglitazone (a PPAR γ agonist) or control medium alone for 24 h. After the designated treatment periods, the culture supernatants were collected, and total intracellular protein was isolated for subsequent biochemical detection and Western blot analysis.

### 2.9. Cellular Immunofluorescence Analysis

Immunofluorescence staining was applied to examine the expression and localization of PPAR-γ in HT-29 cells. Cells were fixed in 4% paraformaldehyde and rinsed with PBS three times (5 min per wash). After permeabilization with 0.1% Triton-X 100 for 10 min, the cells were washed with PBS three times at 5 min intervals. Non-specific binding was blocked with 5% normal goat serum (diluted in PBS) at 37 °C for 1.5 h. The cells were then incubated with primary antibody against PPAR γ (Proteintech, Cat. 16643-1-Ap, 1:250), followed by incubation with Alexa Fluor 594-conjugated goat anti-rabbit secondary antibody (Abcam, Cat. ab150080, 1:400 dilution) at room temperature for 60 min. Cell nuclei were counterstained with DAPI (1 μg/mL), and the samples were washed three times with PBS before observation.

### 2.10. Chromatin Immunoprecipitation Quantitative PCR (ChIP-qPCR)

HT-29 cells were seeded in 10 cm culture dishes at a density of 2 × 10^6^ cells per dish and treated with vehicle, 20 μM rosiglitazone alone, or 10 μM 5-HT combined with 20 μM rosiglitazone for 6 h. Cells were fixed with 1% formaldehyde at room temperature for 10 min, followed by quenching cross-linking with 0.125 M glycine. After washing with pre-cooled PBS, cells were harvested and lysed sequentially with cell lysis buffer and nuclear lysis buffer on ice. Chromatin was fragmented by sonication to yield DNA fragments ranging from 200 to 500 bp. Ten percent of chromatin supernatant was retained as input control.

Equal chromatin fractions were incubated with rabbit anti-PPAR-γ antibody (Proteintech, Cat. 16643-1-Ap, 4 μg per ChIP reaction) or normal rabbit IgG (negative control) at 4 °C overnight. Immunocomplexes were captured using Pierce™ ChIP-grade Protein A/G Magnetic Beads (26162, Thermo Fisher, Waltham, MA, USA). Bead handling strictly followed the manufacturer’s standard protocol without any custom modifications. The bead-chromatin complexes were then sequentially washed with pre-chilled buffers as below:

(1) Low-salt wash buffer: 150 mM NaCl, 20 mM Tris-HCl pH 8.0, 2 mM EDTA, 0.1% SDS, 1% Triton X-100
(2) High-salt wash buffer: 500 mM NaCl, 20 mM Tris-HCl pH 8.0, 2 mM EDTA, 0.1% SDS, 1% Triton X-100
(3) LiCl wash buffer: 0.25 M LiCl, 10 mM Tris-HCl pH 8.0, 1 mM EDTA, 1% NP-40, 1% sodium deoxycholate
(4) TE buffer: 10 mM Tris-HCl pH 8.0, 1 mM EDTA pH 8.0

Bound DNA was eluted using ChIP elution buffer (1% SDS, 100 mM NaHCO_3_). Cross-links were reversed by incubation at 65 °C overnight. Subsequently, samples were digested with RNase A (final concentration 0.2 mg/mL) at 37 °C for 1 h, followed by Proteinase K digestion (final concentration 0.1 mg/mL) at 45 °C for 2 h to remove protein components. DNA was purified with QIAquick PCR Purification Kit (28104, Qiagen, Venlo, The Netherlands).

Quantitative PCR was carried out on a real-time PCR system using PowerTrack™ SYBR Green Master Mix (A46109, Thermo Fisher, Waltham, MA, USA) to amplify two PPRE-containing regions (proximal PPRE, distal PPRE) of the human NLRP6 promoter and an NLRP6 coding negative control fragment. Primer sequences were listed as follows:

Proximal PPRE-F: 5′-TTGCAAAGGTCATGGTGATG-3′

Proximal PPRE-R: 5′-TCATCACCATGACCTTTGCAA-3′

Distal PPRE-F: 5′-ATCAAAGGTCAGAAAACCAAG-3′

Distal PPRE-R: 5′-TCTTTGATTTTCCTTGGTCTC-3′

Negative control-F: 5′-CTGAAGCTGGTGATGAAGAGG-3′

Negative control-R: 5′-GATGGTGGTGATGGTGGTAA-3′

The relative fold enrichment of PPAR-γ was calculated by normalizing the percentage of input DNA from PPAR-γ immunoprecipitation to the IgG control. All experiments were performed in three independent biological replicates with three technical replicates per sample.

### 2.11. Statistical Methods

All data were expressed as mean ± standard error of the mean. Statistical analyses were performed using GraphPad Prism 6.0. The two-tailed independent-samples *t*-test was used for pairwise comparisons between two experimental groups. When multiple independent markers were detected for the same two-group comparison, separate *t*-tests were run for each endpoint, and no multiple-comparison correction was applied for these individual tests. For comparisons involving three or more experimental groups, one-way analysis of variance (ANOVA) followed by Tukey’s post-hoc test was adopted; Tukey’s test was used to adjust *p*-values for multiple pairwise comparisons. A *p* value < 0.05 was considered statistically significant.

## 3. Results

### 3.1. Restraint Stress Increased Intestinal 5-HT Levels

**Figure 1:**
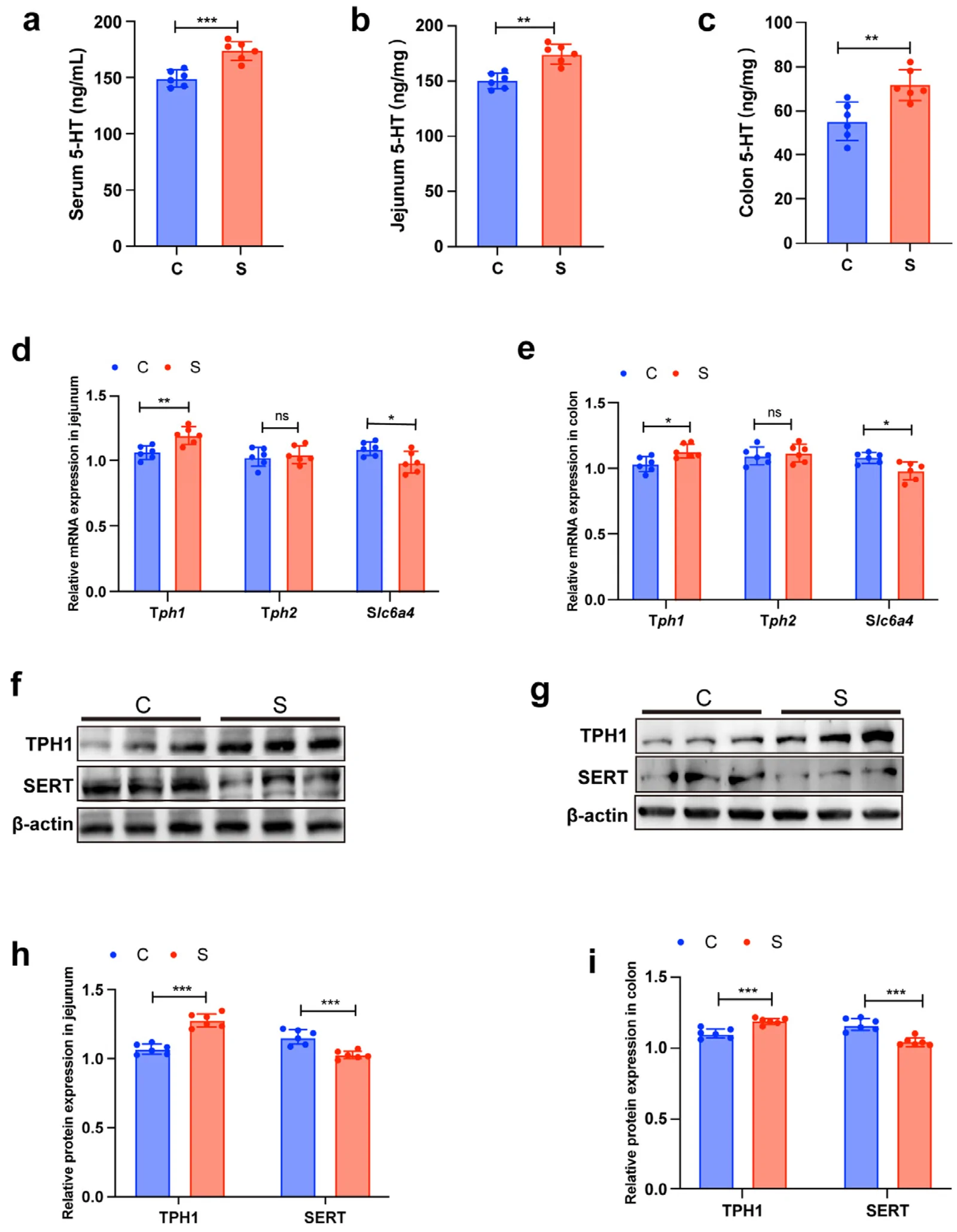
Restraint stress induces an increase in intestinal 5-HT levels in mice. (**a**–**c**) Serum and intestinal tissue concentrations of 5-HT were measured using ELISA (n = 6). (**d**,**e**) The mRNA expression levels of T*ph1*, T*ph2*, and S*lc6a4* in the jejunum and colon were assessed by RT–qPCR. (**f**–**i**) Protein expression of TPH1 and SERT in the jejunum (**f**) and colon (**g**) was evaluated via Western blotting, with β-actin used as the internal control for relative quantification (n = 6; (**h**,**i**)). All data are presented as the mean ± SEM. Statistical significance relative to the control group (C) is indicated as ns: *p* > 0.05,* *p* < 0.05, ** *p* < 0.01, and *** *p* < 0.001. C: control group; S: restraint stress group.

Fourteen consecutive days of restraint stress significantly increased 5-HT levels in serum, jejunum, and colon tissues, as quantified by ELISA (Figure 1a–c). In addition, quantitative polymerase chain reaction (qPCR) and Western blot analysis (Figure 1d–i) revealed a significant increase in the expression of TPH1 in the jejunum and colon tissues of the restraint-stressed mice, accompanied by a significant decrease in SERT expression. This result was consistent with the increase in intestinal 5-HT levels detected by ELISA earlier.

### 3.2. Elevated Intestinal 5-HT Levels Induced Intestinal Mucosal Barrier Destruction

**Figure 2:**
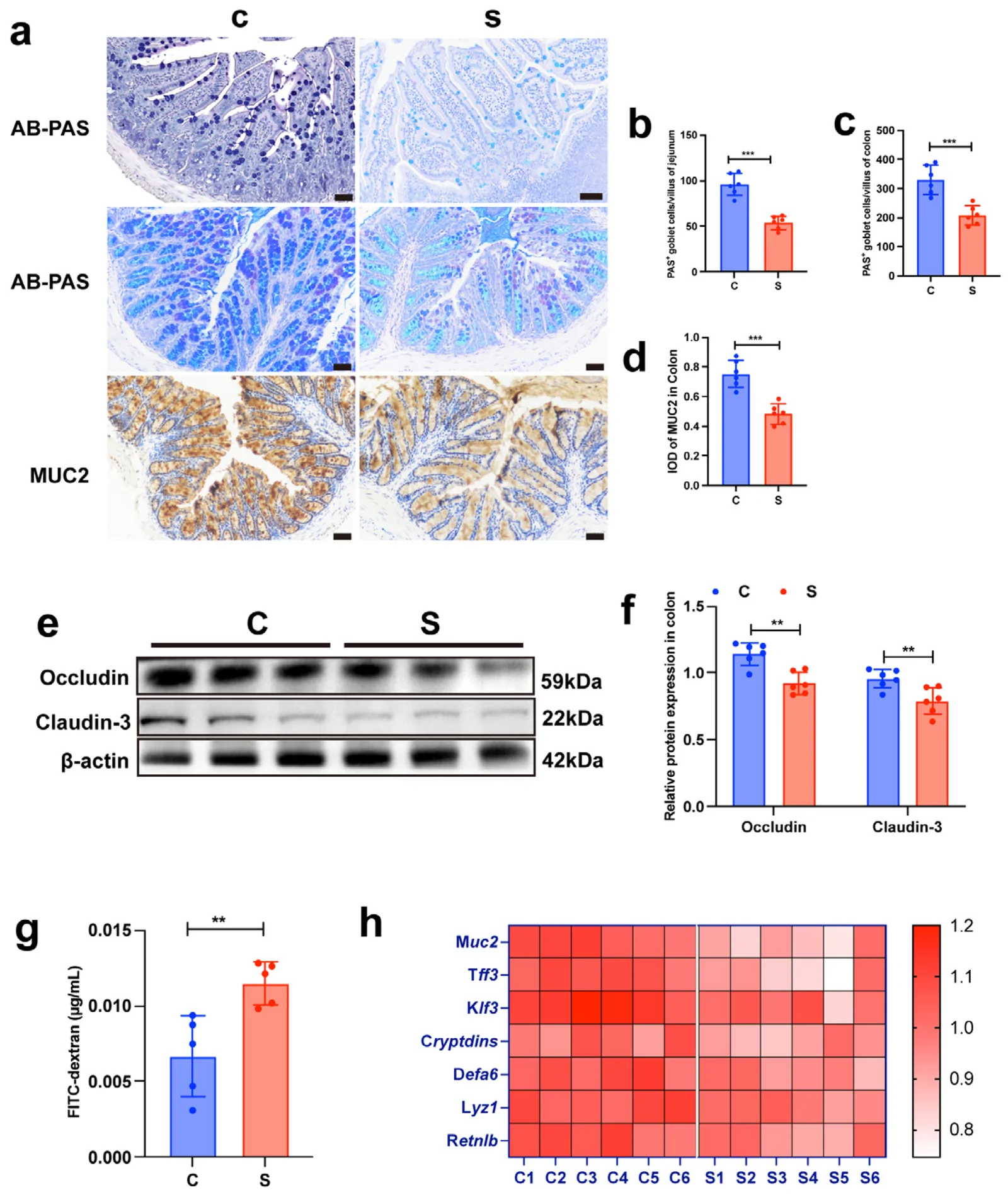
Restraint stress induces disruption of intestinal mucosal barrier. (**a**) AB/PAS staining of jejunal and colonic tissues from mice was performed to visualize goblet cells, with quantification of goblet cell numbers presented in (**b**,**c**). Immunohistochemical staining for MUC2 in colonic tissue is shown in (**a**), and the average integrated optical density (IOD) of positively stained cells was determined using ImageJ (**d**). Scale bars in all staining panels represent 100 μm. (**e**) Protein levels of the tight junction components Occludin and Claudin-3, along with β-actin, were assessed in colonic tissue by Western blotting, and relative expression normalized to β-actin is shown in (**f**) (n = 6). (**g**) Intestinal permeability was evaluated by measuring serum levels of FITC-dextran (n = 6). (**h**) Relative mRNA expression levels of M*uc2* (*p* = 0.0012), T*ff3* (*p* = 0.0021), K*lf3* (*p* = 0.0093), C*ryptdins* (*p* = 0.0432), D*efa6* (*p* = 0.0146), L*yz1* (*p* = 0.0142), and R*etnlb* (*p* = 0.0307) in colon tissue were determined by real-time quantitative PCR (n = 6). All data are expressed as the mean ± SEM. Statistical comparisons to the control group (C) are denoted as ** *p* < 0.01, and *** *p* < 0.001, indicating significant differences. C: control group; S: restraint stress group.

Goblet cells play a critical role in providing a protective mucus barrier covering the gut [5]. Alcian Blue-Periodic Acid-Schiff (AB-PAS) staining of the jejunum and colon revealed that restraint stress significantly reduced the goblet cell number in the intestine (Figure 2a–c). Immunohistochemical staining showed significantly decreased expression of MUC2 secreted by goblet cells following restraint stress stimulation (Figure 2a,d). Tight (Occludin, Claudin-3) junction proteins are known to maintain the integrity of the intestinal mucosal barrier. The Western blot analysis of Occludin and Claudin-3 (Figure 2e,f) both demonstrated significantly inhibited expression of tight junction (Occludin, Claudin-3) proteins following restraint stress treatment. Consistent with this, serum fluorescein isothiocyanate (FITC) levels were significantly increased following restraint stress, indicating increased intestinal permeability (Figure 2g). Furthermore, the expression levels of all seven genes associated with mucosal barrier repair (Muc2, Tff3, Klf3, Cryptdins, Defa6, Lyz1, and Retnlb) were significantly downregulated in the restraint stress group compared with the control group (*p* < 0.05 for each gene) (Figure 2h).

**Figure 3:**
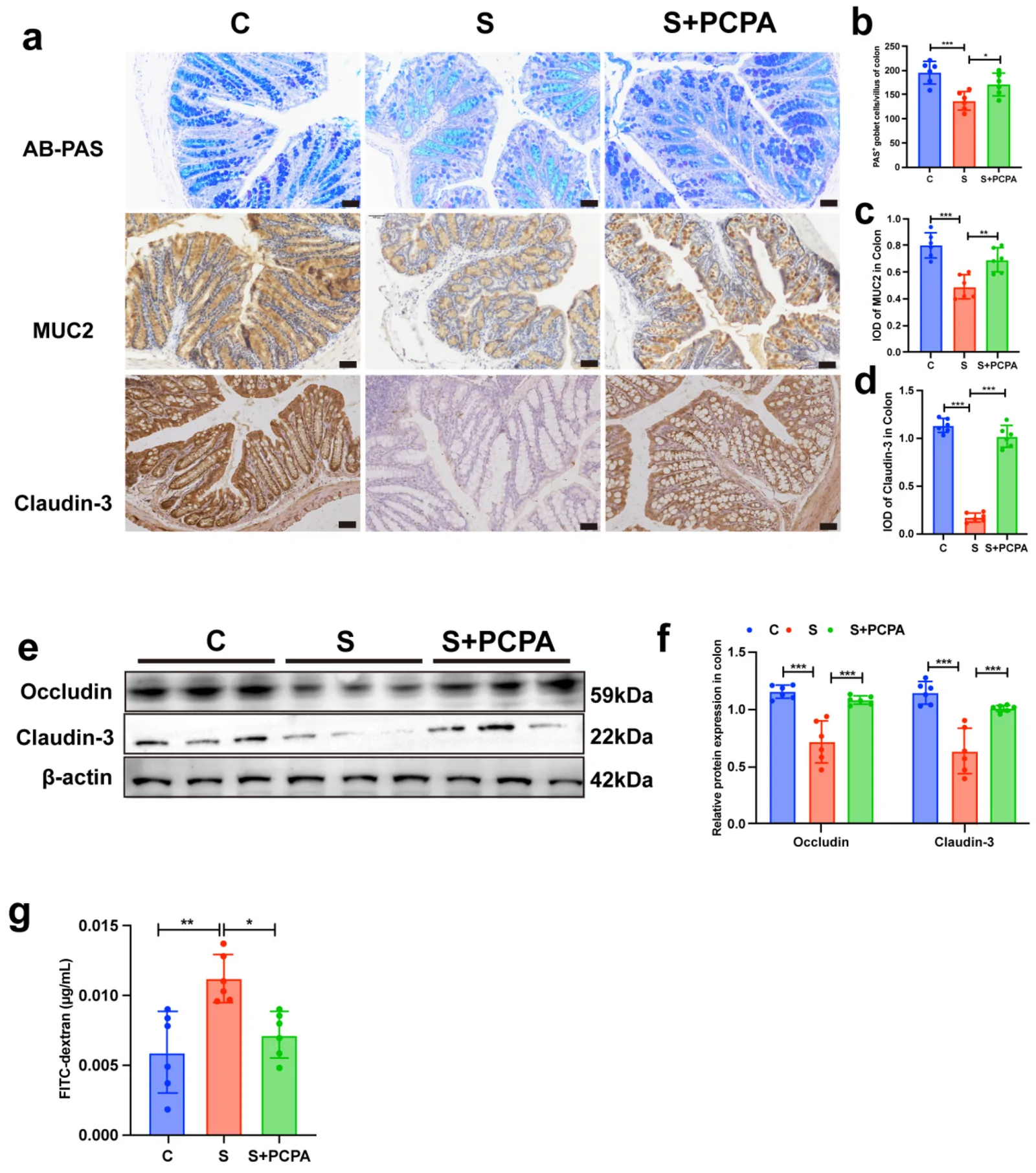
Inhibition of 5-HT production could improve the damage to the intestinal mucosal barrier caused by restraint stress. (**a**) Goblet cells in the colon were visualized using AB/PAS staining, with the corresponding cell counts provided in (**b**). Immunohistochemical staining for MUC2 and Claudin-3 in colonic tissue is also shown in (**a**); the average integrated optical density (IOD) of positive cells was quantified using ImageJ (**c**,**d**). Scale bars in all staining images correspond to 100 μm. (**e**) Protein expression levels of the tight junction proteins Occludin and Claudin-3 in the colon were assessed by Western blotting, with β-actin serving as the loading control. Relative protein levels normalized to β-actin are presented in (**f**) (n = 6). (**g**) Intestinal permeability was evaluated by measuring serum levels of FITC-dextran (n = 6). All data are presented as the mean ± SEM. Statistical significance compared to the control group (C) is indicated as * *p* < 0.05, ** *p* < 0.01, and *** *p* < 0.001. C: control group; S: restraint stress group; S + PCPA: restraint stress plus PCPA group.

To determine whether the destruction of the intestinal mucosal barrier was related to the increase in intestinal 5-HT levels, we intraperitoneally injected restraint-stressed mice with different concentrations of PCPA. The results showed that a high dose of PCPA (150 mg/kg) reduced serum 5-HT levels (Figure S1a) and inhibited TPH1 mRNA expression (Figure S1b). However, the administration of a high dose of PCPA (150 mg/kg) alone did not affect colonic mucosal barrier gene expression (Figure S1d), intestinal permeability (Figure S1e), or MUC2 expression in the colon (Figure S2a,b). Inhibition of 5-HT production in restraint-stressed mice (S + PCPA) alleviated the reduction in goblet cell numbers and MUC2 expression (Figure 3a–c). In the S + PCPA group, both the immunohistochemical staining for Claudin-3 (Figure 3a,d) and Western blot analysis of Occludin and Claudin-3 (Figure 3e,f) showed an upregulation of these tight junction (Occludin, Claudin-3) proteins in the colon. Moreover, the increase in intestinal permeability was also significantly improved (Figure 3g). These results indicated that restraint stress induced an increase in intestinal 5-HT levels, disrupting the intestinal mucosal barrier and leading to an increase in intestinal permeability.

### 3.3. Restraint Stress Attenuated NLRP6 Inflammasome Function and Autophagy Activity

**Figure 4:**
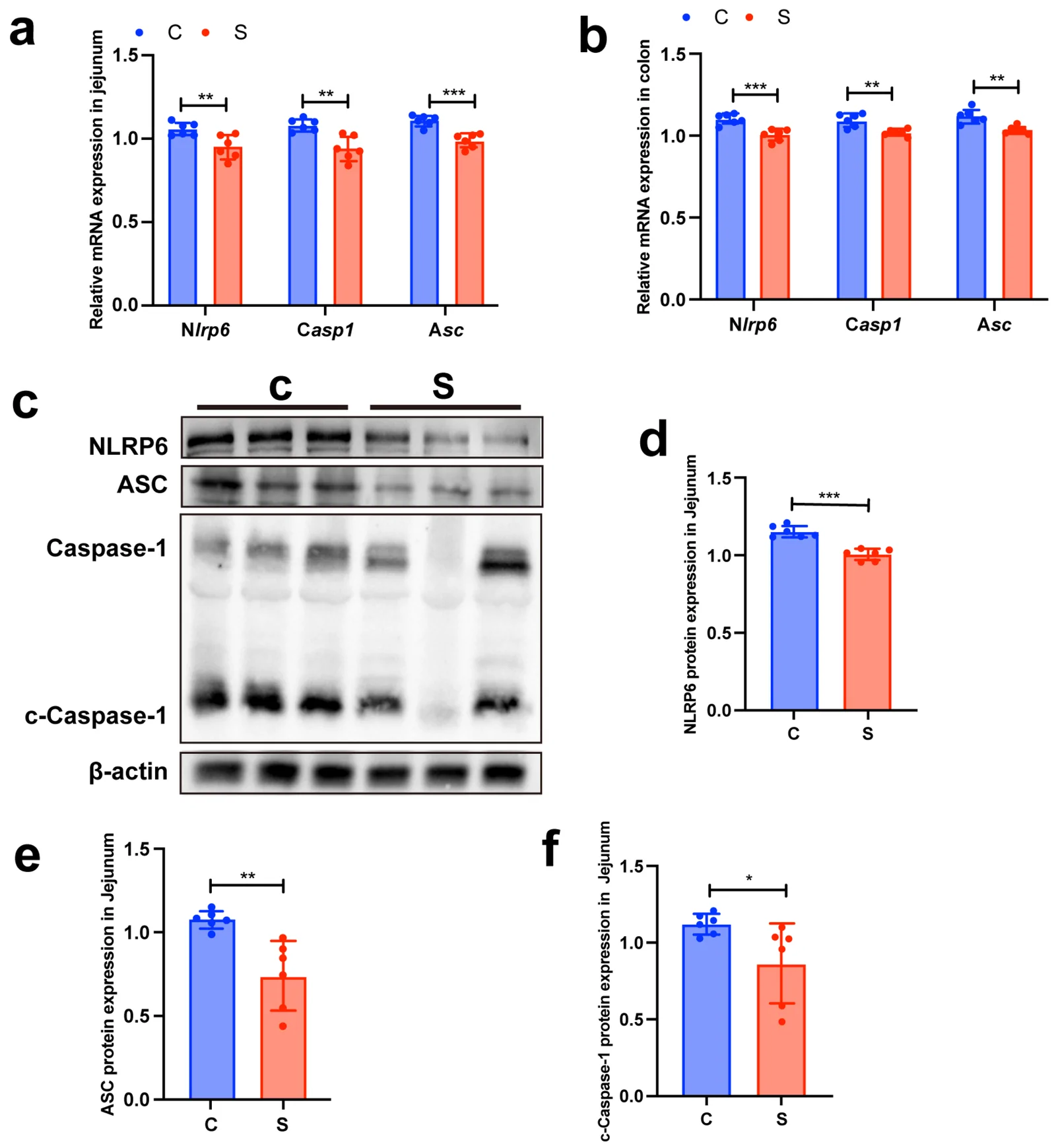
Restraint stress inhibits NLRP6 inflammasome activation. (**a**,**b**) Transcript levels of N*lrp6*, C*asp1*, and A*sc* in the jejunum and colon were quantified using real-time qPCR (n = 6). (**c**) Protein abundance of NLRP6 inflammasome components (NLRP6, cleaved caspase 1, and ASC) in jejunal tissue was assessed by Western blotting, with β actin employed as the loading control. Relative protein levels normalized to β actin are shown in (**d**–**f**) (n = 6). All values are expressed as the mean ± SEM. Statistically significant differences relative to the control group (C) are indicated by * *p* < 0.05, ** *p* < 0.01, and *** *p* < 0.001. C: control group; S: restraint stress group.

As a pattern recognition receptor, NLRP6 was found to regulate MUC2 secretion by goblet cells [14]; we examined the expression of the NLRP6 inflammasome in the intestine. The qPCR results showed that the expression levels of NLRP6 inflammasome–related genes Nlrp6, caspase-1, and Asc were significantly reduced in the jejunum and colon tissues of restraint-stressed mice (Figure 4a,b). The association of NLRP6 and ASC, as well as cleaved caspase-1, represents intracellular activation of the inflammasome. Western blot analysis showed that the protein expression of NLRP6, ASC, and cleaved caspase-1 was significantly lower in the restraint stress group than in the control group (Figure 4c–f).

**Figure 5:**
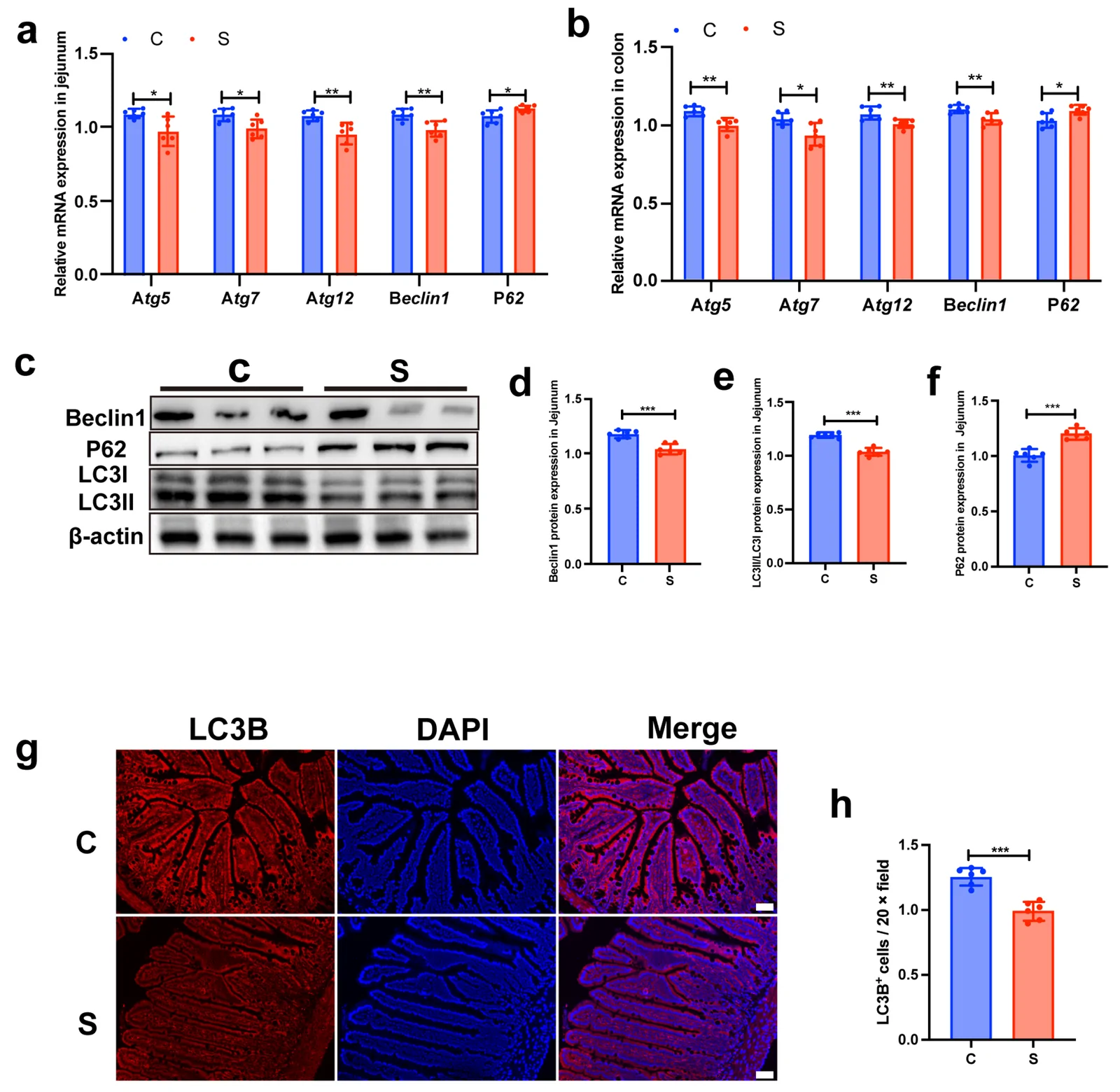
Restraint stress inhibits the occurrence of intestinal autophagy. (**a**,**b**) mRNA levels of autophagy-related genes A*tg5*, A*tg7*, A*tg12*, B*eclin1*, and P*62* in jejunal and colonic tissues were quantified via real-time qPCR (n = 6). (**c**) Protein expression of Beclin1, P62, and the LC3II/LC3I ratio in the jejunum was evaluated by Western blotting, with β actin used as an internal reference. Relative protein abundances normalized to β actin are presented in (**d**–**f**) (n = 6). (**g**) Representative images of LC3B immunofluorescence staining in jejunal tissue are shown, with the scale bar representing 100 μm. (**h**) The intensity of LC3B-positive cells in the jejunum was quantified using ImageJ software. All data are expressed as the mean ± SEM. Statistical differences compared to the control group (C) are denoted as * *p* < 0.05, ** *p* < 0.01, and *** *p* < 0.001. C: control group; S: restraint stress group.

NLRP6 mediates mucosal homeostasis, autophagy, and inflammasome response by controlling the epithelium [16]. qPCR results showed that the expression of autophagy-related genes in the jejunum and colon was significantly downregulated, and P62 expression was significantly upregulated in the restraint stress group (Figure 5a,b). Consistent with these findings, Western blot analysis demonstrated reduced expression of autophagy-related proteins in the intestines of restraint-stressed mice (Figure 5c–f). Furthermore, immunofluorescence staining of the autophagy marker microtubule-associated protein 1A/1B light chain 3B (MAP1LC3B, also known as LC3B) revealed a significant reduction in LC3B expression in the restraint stress group compared with the control group (Figure 5g,h). Collectively, these in vivo data demonstrated that chronic restraint stress suppressed NLRP6 inflammasome expression and significantly impaired intestinal autophagic flux.

### 3.4. Inhibition of 5-HT Production by PCPA Restored the Expression of NLRP6 and Autophagy Proteins in Restraint-Stressed Mice

**Figure 6:**
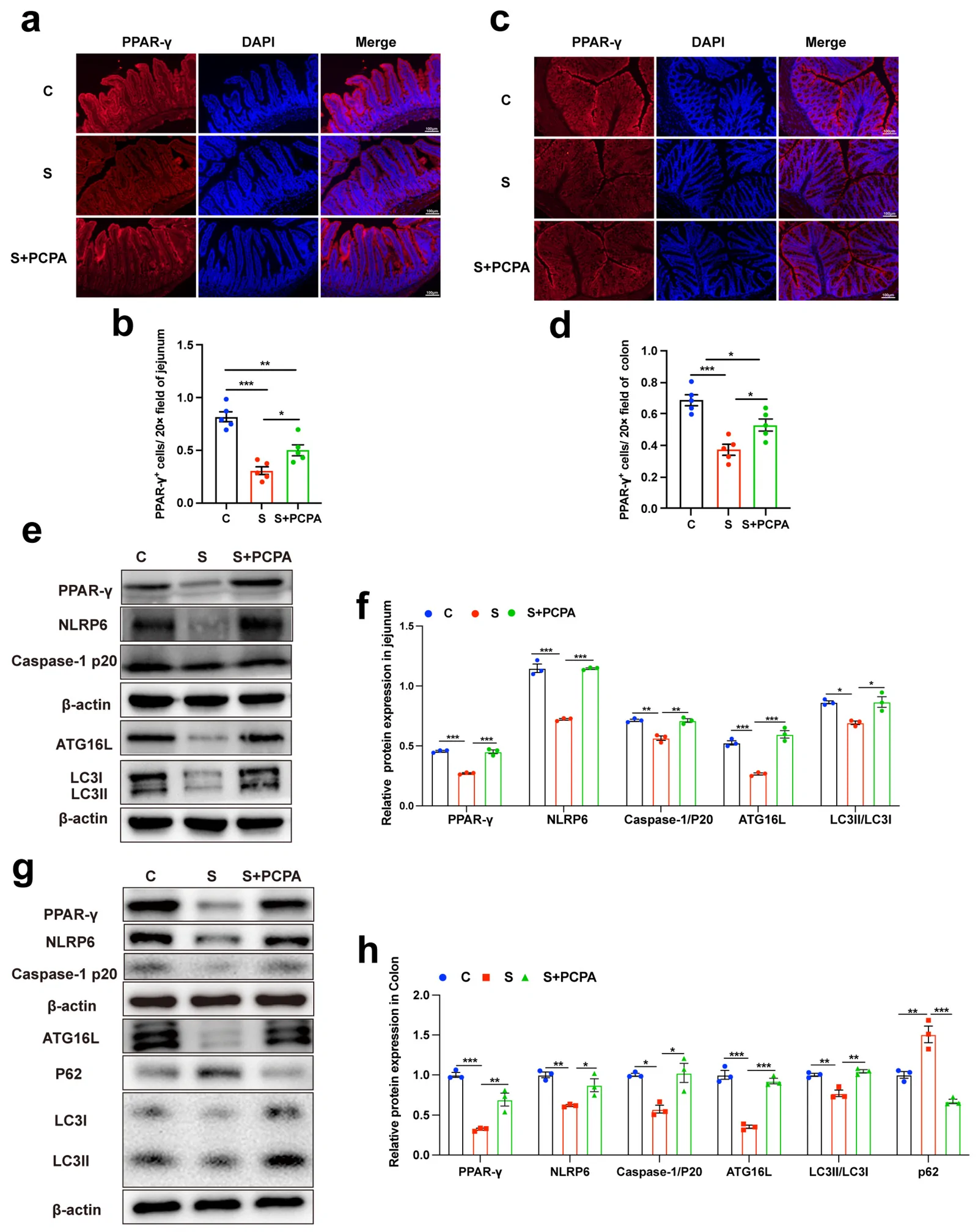
Restraint stress-induced reduction of PPAR-γ, NLRP6 and autophagy protein expression was caused by 5-HT. (**a**,**b**) Immunofluorescence labeling of PPAR-γ in jejunal (**a**) and colonic (**c**) tissues was performed, with representative images shown. The scale bar corresponds to 100 μm. Quantification of PPAR-γ-positive cell intensities in the jejunum (**b**) and colon (**d**) was carried out using ImageJ software. (**e**) Protein levels of PPAR-γ, NLRP6, Caspase-1 p20, ATG16L, and the LC3II/I ratio in jejunal tissue were assessed by Western blotting, with β-actin serving as the loading control. Relative protein expression normalized to β-actin is presented in (**f**) (n = 3). (**g**) In colonic tissue, protein expression of PPAR-γ, NLRP6, Caspase-1 p20, ATG16L, P62, and the LC3II/I ratio was evaluated via Western blotting, using β-actin as an internal reference. Normalized protein levels are shown in (**h**) (n = 3). All values are expressed as the mean ± SEM. Statistical significance relative to the control group (C) is indicated as * *p* < 0.05, ** *p* < 0.01, and *** *p* < 0.001. C: control group; S: restraint stress group; S + PCPA: restraint stress combined with PCPA treatment.

Previous studies have shown that 5-HT inhibits PPAR-γ expression [17]. To determine whether the decrease in PPAR-γ expression was mediated by increased 5-HT levels, we examined the S + PCPA group. Immunofluorescence staining and protein expression showed that restraint stress attenuated the expression of PPAR-γ in the jejunum and colon, whereas PCPA treatment partially restored its expression (Figure 6a–h). In restraint-stressed mice, intraperitoneal injection of PCPA upregulated NLRP6 and cleaved caspase-1 p20 expression (Figure 6e–h). In addition, inhibition of 5-HT production restored the normal expression of autophagy-related proteins Beclin1 and LC3II/I in restraint-stressed mice (Figure 6e–h). These results demonstrated that 5-HT inhibited PPAR-γ expression, which, in turn, affected NLRP6 inflammasome and autophagy expression.

### 3.5. Activation of PPAR-γ Restored NLRP6 Inflammasome, Autophagy, and MUC2 in the Intestine of Restraint-Stressed Mice

**Figure 7:**
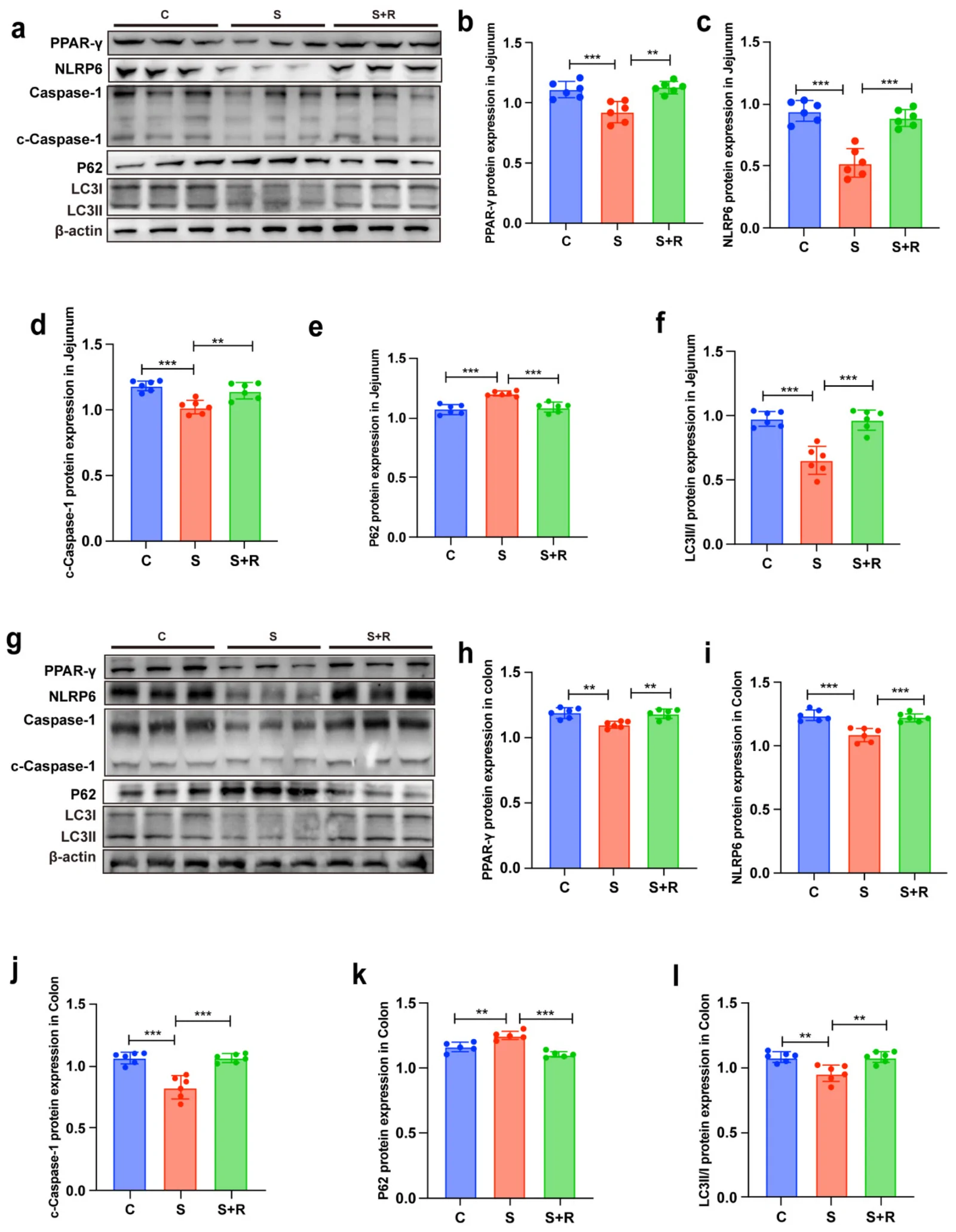
Administration of rosiglitazone in restraint-stressed mice could restore intestinal PPAR-γ and activate the NLRP6 inflammasome and autophagy. (**a**,**g**) Protein expression levels of PPAR-γ, NLRP6, c Caspase 1, P62, and the LC3II/I ratio in jejunal and colonic tissues were assessed by Western blotting. β actin served as the loading control for normalization. Relative protein abundances of PPAR-γ, NLRP6, cleaved Caspase-1, p62, and the LC3-II/I ratio in the jejunum are shown in panels (**b**–**f**), while those in the colon are presented in panels (**h**–**l**) (n = 6). All data are expressed as the mean ± SEM. Statistical significance compared with the control group (C) is indicated by ** *p* < 0.01 and *** *p* < 0.001. C: control group; S: restraint stress group; S + R: restraint stress plus Rosiglitazone group.

In terms of the transcriptional regulation of NLRP6, PPAR-γ binding sites were identified in the promoter region of NLRP6 [18]. PPAR-γ agonist rosiglitazone has been shown to upregulate NLRP6 in vivo [13]. To further investigate the influence of PPAR-γ on the NLRP6 inflammasome, restraint-stressed mice were initially administered different concentrations of rosiglitazone through oral gavage. The results revealed that a high dose of rosiglitazone (30 mg/kg) upregulated the expression of PPAR-γ (Figure S1c). Furthermore, a high dose of rosiglitazone (30 mg/kg) administered alone did not alter the expression of genes related to the colonic mucosal barrier (Figure S1d), intestinal permeability (Figure S1e), or MUC2 secretion in the colon (Figure S2a,b), as demonstrated by histological staining. However, Western blot analysis showed that rosiglitazone administration restored the expression of PPAR-γ, activated the NLRP6 inflammasome, and restored the ability of intestinal autophagy in restraint-stressed mice (Figure 7).

**Figure 8:**
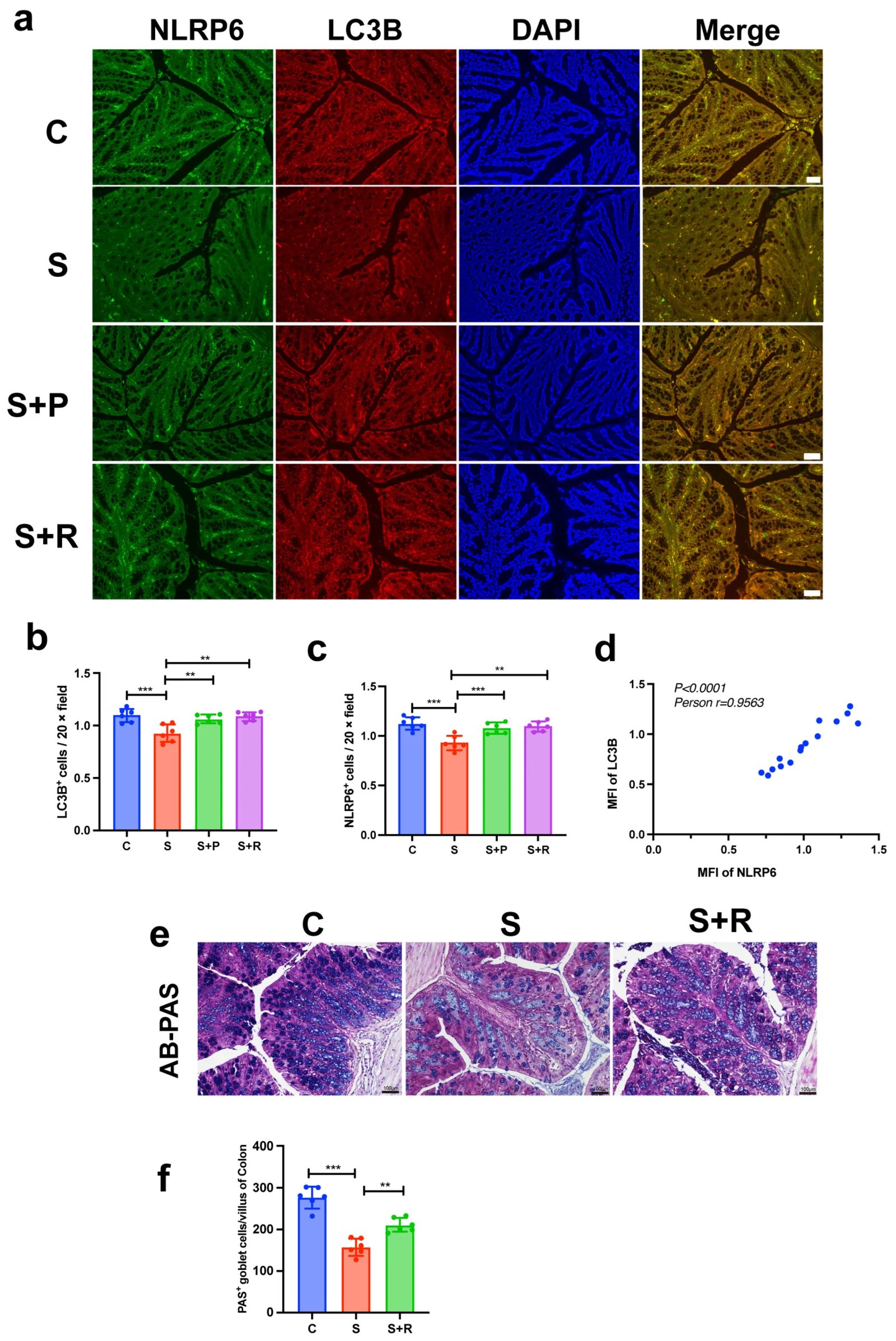
Rosiglitazone could activate the expression of NLRP6 inflammasome and autophagy, and restore the number of goblet cells and mucin secretion in restraint stress mice. (**a**) Co-localization of NLRP6 and LC3B in colonic tissue was examined by immunofluorescence staining, with representative images provided in (**a**). (**b**,**c**) Fluorescence intensities of NLRP6-positive and LC3B-positive cells in the colon were quantified using ImageJ software, and the results are shown in (**b**,**c**), respectively. (**d**) The mean fluorescence intensity (MFI) of NLRP6 (X-axis) and LC3B (Y-axis) was quantified within each region of interest (ROI). Each data point corresponds to a single ROI rather than individual image pixels. X-axis: MFI of NLRP6 (arbitrary units, a.u.); Y-axis: MFI of LC3B (arbitrary units, a.u.). (**e**) Goblet cells in the colon were visualized by AB/PAS staining, and their numbers were quantified; both the representative images and the quantification are displayed in (**f**). Scale bars in all images correspond to 100 μm. All data are expressed as the mean ± SEM. Statistically significant differences relative to the control group (C) are indicated as ** *p* < 0.01 and *** *p* < 0.001. C: control group; S: restraint stress group; S + R: restraint stress plus Rosiglitazone group.

To further verify whether PPAR-γ activation promotes NLRP6 inflammasome activation in the intestine and enhances autophagy, immunofluorescence co-staining was performed to observe the co-localization of NLRP6 and LC3B in colonic tissues. Restraint stress reduced the number of cells positive for NLRP6 and LC3B co-localization (Figure 8a–c). In contrast, inhibition of 5-HT synthesis in restraint-stressed mice significantly increased the number of cells exhibiting NLRP6 and LC3B co-localization (Figure 8a–c). Additionally, PPAR-γ activation by rosiglitazone also markedly increased the number of cells positive for NLRP6 and LC3B co-localization in restraint-stressed mice (Figure 8a–c). To enable quantitative analysis of PPAR-γ and LC3B co-localization, Pearson’s correlation coefficient (PCC) analysis was performed on the immunofluorescence results. PCC quantifies the linear correlation between the spatial distributions of the two protein signals based on pixel intensity, with an r-value approaching 1 indicating a higher degree of co-localization between NLRP6 and LC3B (Figure 8d). Notably, the addition of rosiglitazone alleviated the reduction in colonic goblet cell numbers caused by restraint stress (Figure 8e,f). These results indicated that PPAR-γ activation restored NLRP6 inflammasome expression, enhanced the expression of autophagy-related proteins, and improved the restraint stress–induced damage of the intestinal mucosal barrier.

### 3.6. 5-HT Attenuated NLRP6 Inflammasome Function and Autophagy Level by Inhibiting Intestinal PPAR-γ Expression

**Figure 9:**
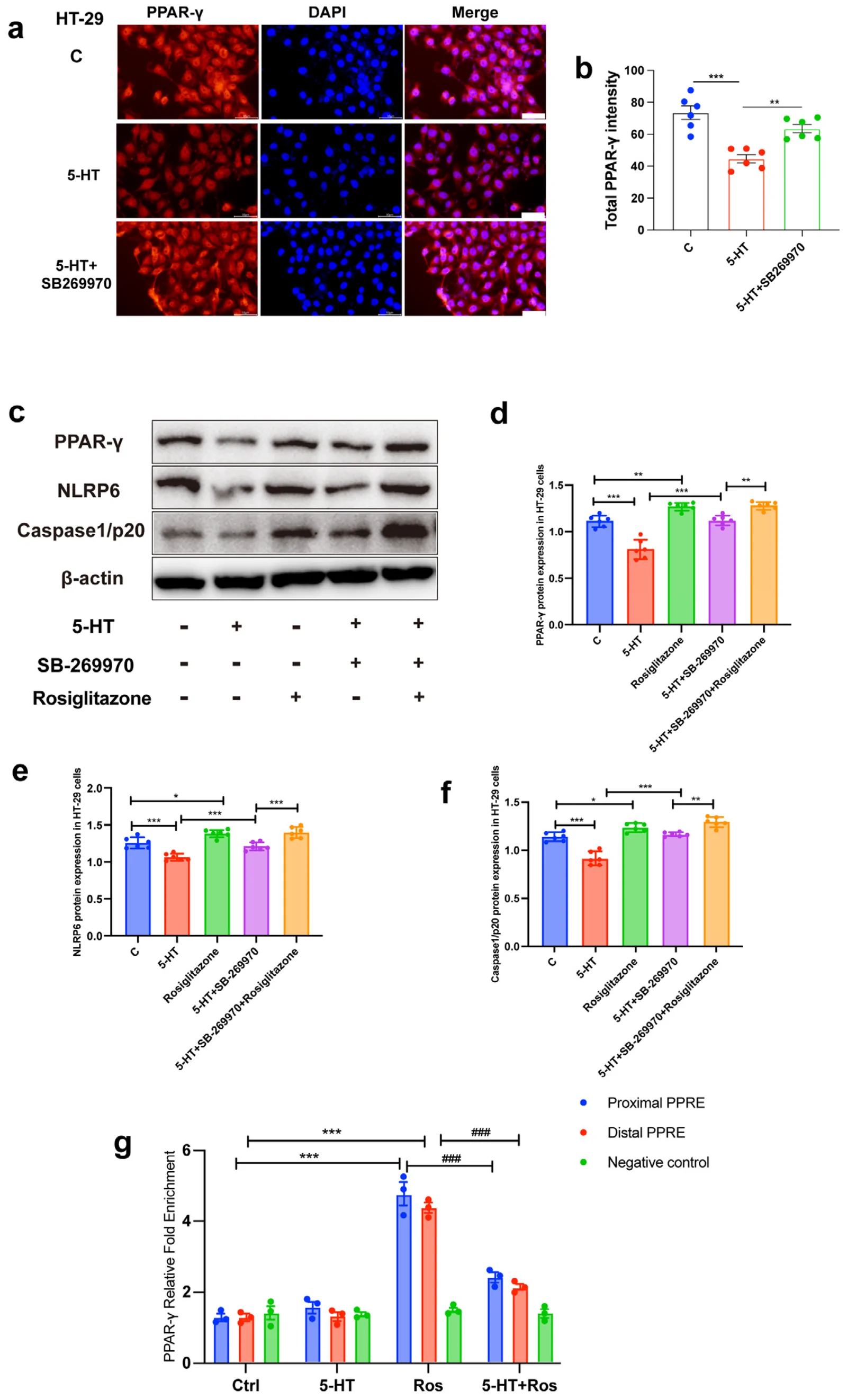
5-HT inhibited the expression of PPAR-γ through HTR7 receptor, thereby disrupting the function of NLRP6. (**a**) Immunofluorescence visualization of PPAR-γ in HT-29 cells is shown, with representative images presented with a scale bar = 100 μm. Fluorescence intensity of PPAR-γ was quantified using ImageJ software and is summarized in (**b**) (n = 6 wells from 6 independent biological experiments). (**c**) HT-29 cells were subjected to treatment with 5-HT, the HTR7 antagonist SB269970, or rosiglitazone, either alone or in combination. Protein levels of PPAR-γ, NLRP6, and Caspase-1 p20 were subsequently assessed by Western blotting, with β-actin serving as the loading control. Relative protein expression normalized to β-actin is presented in (**d**–**f**) (n = 6 wells from 6 independent biological experiments). (**g**) ChIP-qPCR analysis of PPAR-γ binding to NLRP6 promoter. PPAR-γ enrichment at proximal and distal PPRE sites was quantified in HT-29 cells treated with vehicle, rosiglitazone, or rosiglitazone combined with 5-HT. The NLRP6 coding region was used as negative control. All data are reported as the mean ± SEM. Statistically significant differences relative to the control group (C) are denoted as * *p* < 0.05, ** *p* < 0.01, and *** *p* < 0.001. ### *p* < 0.001 vs. rosiglitazone group.

**Figure 10:**
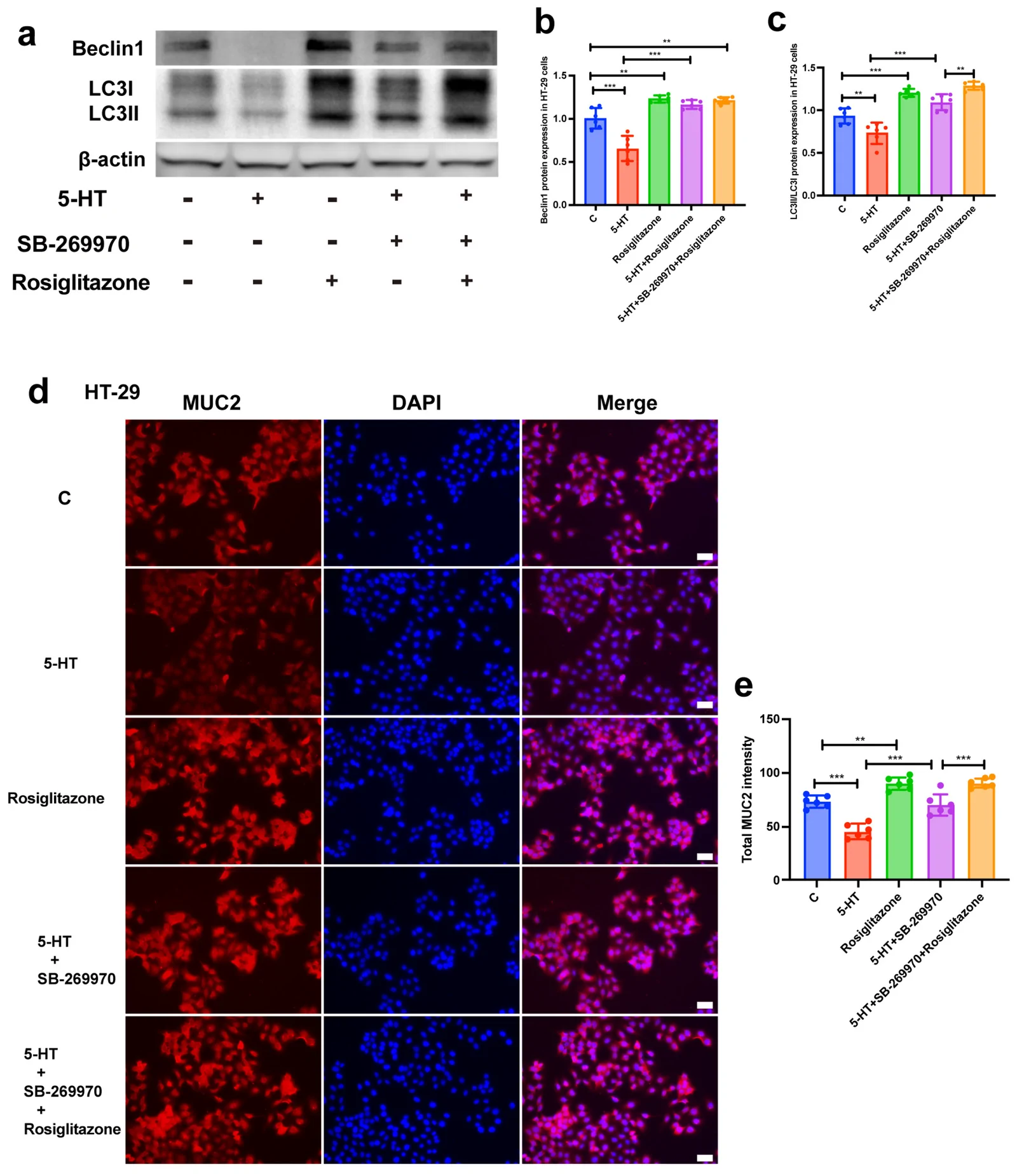
5-HT inhibited the expression of PPAR-γ through HTR7 receptor, thereby disrupting the expression of autophagy and MUC2 proteins. (**a**) Western blot analysis was performed to determine the protein abundance of Beclin1 and the LC3II/LC3I ratio in HT-29 cells following treatment with 5-HT, the HTR7 antagonist SB269970, or rosiglitazone. β-actin was used as an internal control for normalization, with relative protein levels shown in (**b**,**c**) (n = 6 wells from 6 independent biological experiments). (**d**) Immunofluorescence staining was employed to visualize MUC2 expression in HT-29 cells, and representative images are presented in (**d**) (scale bar = 100 μm). Fluorescence intensity of MUC2 was quantified using ImageJ software, with results summarized in (**e**) (n = 6 wells from 6 independent biological experiments). All experimental data are expressed as the mean ± SEM. Statistical comparisons to the control group (C) are indicated by ** *p* < 0.01, and *** *p* < 0.05, denoting significant differences.

To further confirm the role of 5-HT in inhibiting PPAR-γ expression, thereby suppressing NLRP6 inflammasome activation, blocking autophagy, and disrupting the intestinal mucosal barrier, we validated the regulatory cascade in HT-29 cells. Consistent with in vivo observations, immunofluorescence staining revealed that 5-HT suppressed PPAR-γ protein expression, whereas 2 h pretreatment with the HTR7 antagonist SB-269970 reversed this inhibitory effect (Figure 9a,b). Western blot analysis further demonstrated that 5-HT downregulated the expression of PPAR-γ, NLRP6, and cleaved caspase-1 p20, whereas SB-269970 co-treatment rescued their expression (Figure 9c–f). ChIP-qPCR was performed to verify the direct interaction between PPAR-γ and the NLRP6 promoter. Rosiglitazone significantly enhanced PPAR-γ enrichment at both the proximal and distal PPRE motifs within the NLRP6 promoter (Figure 9g). In contrast, co-administration of 5-HT abrogated rosiglitazone-mediated PPAR-γ recruitment (Figure 9g). No specific PPAR-γ binding was detected at the NLRP6 coding negative control region across all groups (Figure 9g). These findings indicated that 5-HT repressed PPAR-γ transcription through the HTR7 receptor to reduce NLRP6 expression. Notably, combined treatment with SB-269970, 5-HT, and rosiglitazone markedly upregulated PPAR-γ, NLRP6, and caspase-1 p20 (Figure 9c–f), further confirming that PPAR-γ activation directly drives NLRP6 expression. 5-HT stimulation markedly reduced autophagic activity, as evidenced by decreased Beclin1 expression and a lower LC3-II/LC3-I ratio, whereas SB-269970 reversed these changes (Figure 10a–c), suggesting that 5-HT suppresses autophagy via HTR7. Compared with the SB-269970 + 5-HT group, additional rosiglitazone further elevated Beclin1 and LC3II/I levels (Figure 10a–c), supporting the role of PPAR-γ activation in promoting autophagy. Given that HT-29 cells constitutively secrete the mucosal protective MUC2, we further investigated the role of the 5-HT/HTR7/PPAR-γ axis in mucin secretion. Exposure to 5-HT inhibited MUC2 secretion, whereas SB-269970 restored its expression (Figure 10d,e). Moreover, rosiglitazone alone or co-treatment with SB-269970 + 5-HT robustly boosted MUC2 release (Figure 10d,e). Collectively, PPAR-γ activation restored NLRP6 inflammasome function and autophagic flux, thereby promoting MUC2 secretion and preserving intestinal mucosal barrier integrity.

## 4. Discussion

Previous studies have independently demonstrated associations among 5-HT/HTR7, PPAR-γ, NLRP6 inflammasome, autophagy, and goblet cell mucus secretion. However, no study has constructed a complete linear molecular cascade under psychological stress to interpret intestinal mucosal barrier damage or clarified the subtype-specific clinical applicability of this signaling pathway for IBS. Clinically, IBS is classified into four subtypes: diarrhea-predominant IBS (IBS-D), constipation-predominant IBS (IBS-C), mixed IBS (IBS-M), and unclassified IBS (IBS-U), each exhibiting distinct intestinal 5-HT metabolic phenotypes [2,19]. Our 14-day restraint stress mouse model successfully recapitulated key pathological characteristics of IBS-D, including excessive peripheral intestinal 5-HT accumulation, reduced goblet cell numbers, impaired MUC2 secretion, and increased intestinal permeability [4,20]. In contrast, IBS-C is characterized by decreased intestinal 5-HT synthesis and release [2,19]. Therefore, the 5-HT/HTR7/PPAR-γ/NLRP6/autophagy axis identified in this study cannot be generalized to IBS-C, IBS-M, or IBS-U subtypes. This subtype-specific limitation of the pathway provides precise theoretical support for subtype-targeted clinical interventions in IBS, rather than for universal 5-HT-targeted therapies across all IBS subtypes.

Clinical evidence has demonstrated that the intestinal 5-HT signaling pathway—encompassing EC cells, 5-HT synthesis, release, SERT-mediated reuptake, and receptor signaling—is significantly altered in patients with IBS and inflammatory bowel disease (IBD) [2,19,21,22,23,24]. These alterations directly affect gastrointestinal motility, secretion, and visceral sensation, contributing to the pathogenesis and clinical manifestations of these disorders. Consequently, 5-HT and its receptors have emerged as important therapeutic targets for functional gastrointestinal disorders and IBD [19,25]. In the present study, restraint stress markedly elevated serum, jejunal, and colonic 5-HT levels, accompanied by significant upregulation of intestinal EC cell-specific TPH1 and downregulation of SERT, whereas brain/neuronal TPH2 expression remained unchanged. These findings indicate that stress-induced intestinal 5-HT accumulation is predominantly derived from mucosal EC cells rather than enteric neurons, consistent with previous studies showing increased abnormal EC cells and elevated 5-HT levels in patients with IBD and IBS [26]. Consistently, Tph1-knockout mice displayed decreased intestinal 5-HT levels and alleviated chemically induced colitis [27], whereas elevated intestinal 5-HT exacerbated intestinal inflammation in IL-10-deficient colitis mice, further verifying the role of 5-HT as a pivotal pathogenic signal driving intestinal mucosal dysfunction [28].

Intestinal hyperpermeability is a hallmark pathological feature of IBS [20]. The present study demonstrated that restraint stress triggered severe intestinal mucosal barrier disruption, as evidenced by reduced goblet cell numbers, suppressed MUC2 secretion, decreased tight junction (Occludin, Claudin-3) expression, and global downregulation of mucosal repair-related genes (Muc2, Tff3, Klf3, Cryptdins, Defa6, Lyz1, and Retnlb). MUC2 secreted by goblet cells forms the inner sterile mucus layer to block microbial translocation [29], and Muc2-deficient mice spontaneously develop colitis. Meanwhile [30], patients with ulcerative colitis exhibit impaired MUC2 synthesis and disruption of the mucus layer [31]. Pharmacological inhibition of 5-HT synthesis through high-dose PCPA largely reversed the stress-induced intestinal mucosal barrier defects, directly proving that excess intestinal 5-HT under stress is the upstream trigger of goblet cell dysfunction and leaky gut.

NLRP6 inflammasome is a key NLR family sensor maintaining intestinal epithelial homeostasis. It recruits ASC to activate caspase-1/11, releasing IL-1β and IL-18 to regulate mucosal self-renewal, inflammation, and mucus secretion [14]. Previous studies using the WAS model have confirmed that stress inhibits NLRP6 inflammasome activation, inducing small intestinal lesions [13]. Although NLRP6-knockout mice exhibit goblet cell hyperplasia, they display defective mucus granule secretion and a discontinuous inner mucus layer [14]. Mechanistically, NLRP6 inflammasome activity is indispensable for goblet cell autophagy, and NLRP6 deletion severely impairs intestinal epithelial autophagy flux [14,16]. Prior studies have also reported that excess intestinal 5-HT represses autophagy through mTOR signaling, thereby increasing colitis susceptibility [32]. Consistent with these findings, our in vivo data demonstrated that restraint stress suppressed the transcription and protein expression of NLRP6, ASC, and cleaved caspase-1, accompanied by downregulation of autophagy-related genes (Atg5, Atg7, Atg12, and Beclin1), accumulation of autophagy substrate P62, and reduction of LC3B puncta in intestinal epithelium. These alterations were largely reversed by PCPA-mediated 5-HT depletion, indicating that stress-induced 5-HT overproduction simultaneously suppresses NLRP6 inflammasome and autophagy, thereby jointly impairing goblet cell mucus secretion.

We further investigated the transcriptional bridge linking 5-HT and NLRP6/autophagy: PPAR-γ. Previous bioinformatic analysis and functional validation have confirmed the presence of functional PPAR-γ response elements within the NLRP6 promoter, and PPAR-γ agonists have been shown to exert anti-inflammatory and intestinal mucosa-protective effects by transcriptionally elevating NLRP6 levels [18]. To provide direct molecular evidence for this transcriptional interaction, we performed ChIP-qPCR in colorectal adenocarcinoma HT-29 cells and confirmed the physical binding of PPAR-γ to the NLRP6 promoter. Functional assays further demonstrated that rosiglitazone robustly increased PPAR-γ enrichment at the proximal and distal PPRE motifs of the NLRP6 promoter, whereas 5-HT co-treatment markedly abolished rosiglitazone-mediated PPAR-γ recruitment, explaining the transcriptional repression of NLRP6 following 5-HT stimulation. Colonic epithelial PPAR-γ expression is significantly reduced in patients with ulcerative colitis, and PPAR-γ signaling restrains pathobiont overgrowth while regulating small intestinal mucosal immune defense [33]. Consequently, PPAR-γ has emerged as a promising therapeutic target for IBD. Consistent with the WAS model data that rosiglitazone reverses stress-induced NLRP6 downregulation [18], our in vivo drug intervention demonstrated that both PCPA and rosiglitazone restored PPAR-γ, NLRP6 inflammasome, and autophagy protein expression in restraint-stressed mice.

In vitro HT-29 cells experiment further verified the complete receptor-dependent axis. Specifically, 5-HT suppressed PPAR-γ expression, whereas pretreatment with the HTR7 antagonist SB-269970 completely abrogated this inhibitory effect, confirming that 5-HT signals through HTR7 to repress PPAR-γ. This observation was consistent with previous studies reporting that the 5-HT/HTR7 axis inhibits colonic epithelial β-defensin production through PPAR-γ downregulation [17]. Additionally, 5-HT treatment suppressed NLRP6, cleaved caspase-1, and autophagy markers (Beclin1 and LC3II/I ratio), which were restored by SB-269970. Treatment with SB-269970 + 5-HT + rosiglitazone further boosted NLRP6 and autophagy protein levels, supporting the role of PPAR-γ as an upstream transcriptional activator of NLRP6. Notably, rosiglitazone could not directly modulate the autophagy machinery; rather, PPAR-γ-dependent NLRP6 upregulation secondarily restored autophagy flux, consistent with previous reports that NLRP6 deficiency intrinsically blocks goblet cell autophagy [14]. Immunofluorescence co-localization of NLRP6 and LC3B in colon tissues further validated this synergistic regulatory relationship, and Pearson correlation coefficient quantification confirmed that restraint stress reduced NLRP6-LC3B co-localization, which was reversed by PCPA or rosiglitazone. Under basal conditions, HT-29 cells secrete MUC2, whereas exogenous 5-HT significantly inhibits MUC2 secretion. Treatment with either SB-269970 or rosiglitazone restored MUC2 secretion, thereby directly linking the 5-HT/HTR7/PPAR-γ/NLRP6/autophagy signaling pathway to the core functional deficits of goblet cells under psychological stress. Collectively, this study innovatively constructed a full downstream signaling branch of the canonical 5-HT/HTR7/PPAR-γ axis, revealing one plausible mechanistic cascade (rather than the sole pathway) underlying stress-triggered intestinal mucosal barrier injury. Beyond this epithelial-intrinsic cascade, stress-induced microbiota perturbation represents another well-documented contributor to gut barrier dysfunction.

## 5. Conclusions

In summary, this study verified the established 5-HT/HTR7/PPAR-γ regulatory axis in intestinal tissues. We further demonstrated that this pathway mediated restraint stress–induced inhibition of NLRP6 inflammasome and autophagy, leading to impaired goblet cell function and disruption of the intestinal mucosal barrier. This extended signaling network revealed a previously unreported mechanism underlying stress-related intestinal injury.

## 6. Study Limitations

This study has several noteworthy limitations for translational interpretation. First, all in-vivo animal experiments were performed exclusively in female C57BL/6J mice; given known sex-based disparities in irritable bowel syndrome prevalence [34], the observed regulatory cascade may not fully recapitulate signaling events in male organisms. Second, mechanistic evidence largely relies on pharmacological interventions using PCPA and SB-269970, which carry potential off-target effects; future genetic-editing studies are required to confirm cell-type-specific functions of this signaling axis. Most importantly, our conclusions are derived from mouse restraint-stress models and HT-29 colorectal adenocarcinoma cells rather than primary human intestinal epithelial tissues. HT-29 is a cancer-derived cell line with intrinsic phenotypic deviations from normal human gut epithelium, and mouse intestinal physiology also differs from that of humans. Therefore, direct extrapolation of these mechanistic observations to human intestinal pathophysiology should be done with caution, and further clinical validation using human intestinal biopsy samples from patients with diarrhea-predominant irritable bowel syndrome is essential to verify the clinical relevance of the 5-HT/HTR7/PPAR-γ/NLRP6/autophagy pathway in humans.

## Data Availability

The original contributions presented in this study are included in the article/Supplementary Materials. Further inquiries can be directed to the corresponding author.

## Supplementary Materials

The following supporting information can be downloaded at: https://www.mdpi.com/article/10.3390/biology15201870/s1, Figure S1. Verification of Drug Concentration Results. Figure S2. Colonic Histological Staining Results. Figure S3. Original Western blot images for Figure 1, Figure 2, Figure 3, Figure 4, Figure 5, Figure 6, Figure 7, Figure 9 and Figure 10. Table S1. Primers for real-time PCR. Table S2. Antibodies used in this study.

## Abbreviations

The following abbreviations are used in this manuscript:

5-H	5-Hydroxytryptamine
EC	Enterochromaffin Cell
TPH1	Tryptophan Hydroxylase 1
SERT	Serotonin-Selective Reuptake Transporter
MUC2	Mucin 2
NLRP6	NOD-Like Receptor Family Pyrin Domain Containing 6
ASC	Apoptosis-Associated Speckle-Like Protein
PPAR-γ	Peroxisome Proliferator-Activated Receptor Gamma
FITC	Fluorescein Isothiocyanate
HTR7	5-Hydroxytryptamine Receptor 7
LC3B	Microtubule-Associated Protein 1A/1B Light Chain 3B
