# Supplementary material for: Psychological Stress Disrupts Goblet Cell Function and Intestinal Barrier via 5-HT/HTR7-Mediated NLRP6 and Autophagy Suppression
Source: Biology (Basel). 2026 Oct 16;15(20):1870. doi: 10.3390/biology15201870 (PMC13701723; doi:10.3390/biology15201870)
Supplement: Supplementary file 1 [file biology-15-01870-s001.zip › biology-4599106-supplementary.pdf]

Supplementary Material

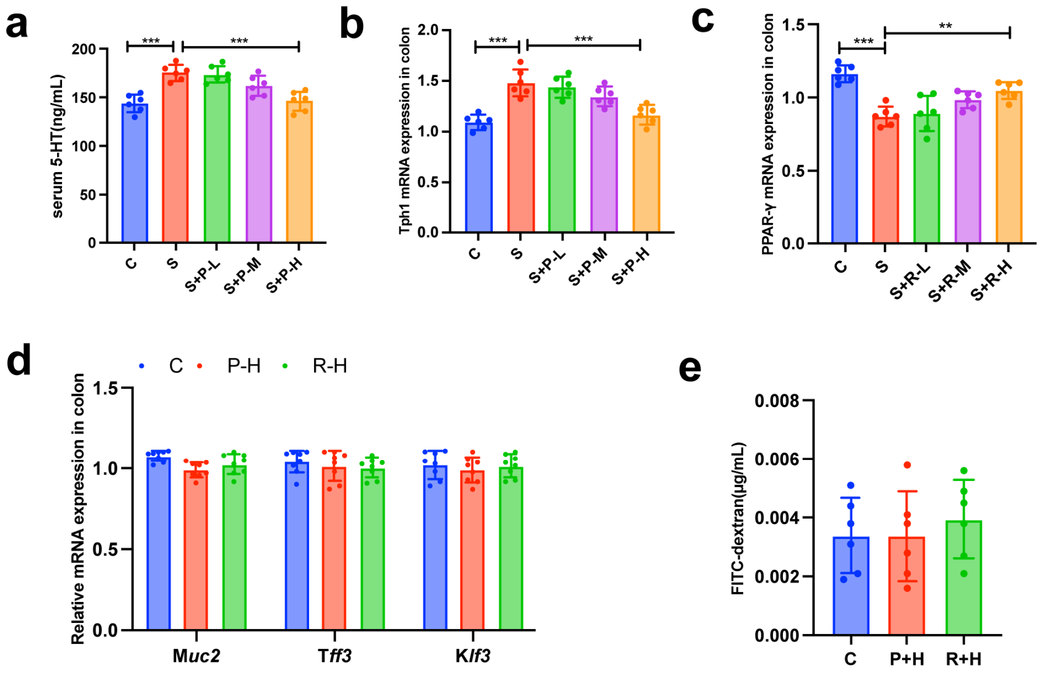

Figure S1 Verification of Drug Concentration Results

(a) Serum 5-HT levels were measured by ELISA. (b) qPCR was used to detect the change of T*ph1*mRNA level in colon tissues. (c) qPCR was used to detect the change of P*PAR-γ* mRNA level in colon tissues. (d) qPCR was used to detect the change of M*uc2*, T*ff3* and K*lf3* mRNA level in colon tissues. (e) Intestinal permeability was evaluated by measuring serum levels of FITC-dextran (n = 6). Control group (C; n =6), Restraint stress group (S; n =6), Restraint stress +PCPA group: S+P-L (50 mg/kg), n=6; S+P-M (100 mg/kg), n=6; S+P-H (150 mg/kg), n=6; Restraint stress + Rosiglitazone group: S+P-L (10 mg/kg), n=6; S+P-M (20 mg/kg), n=6; S+P-H (30 mg/kg), n=6; PCPA group: P-H (150 mg/kg), n=6; Rosiglitazone group: R-H (30 mg/kg), n=6. Data are presented as the mean ± SEM. $**P < 0.01$; $***P < 0.001$.

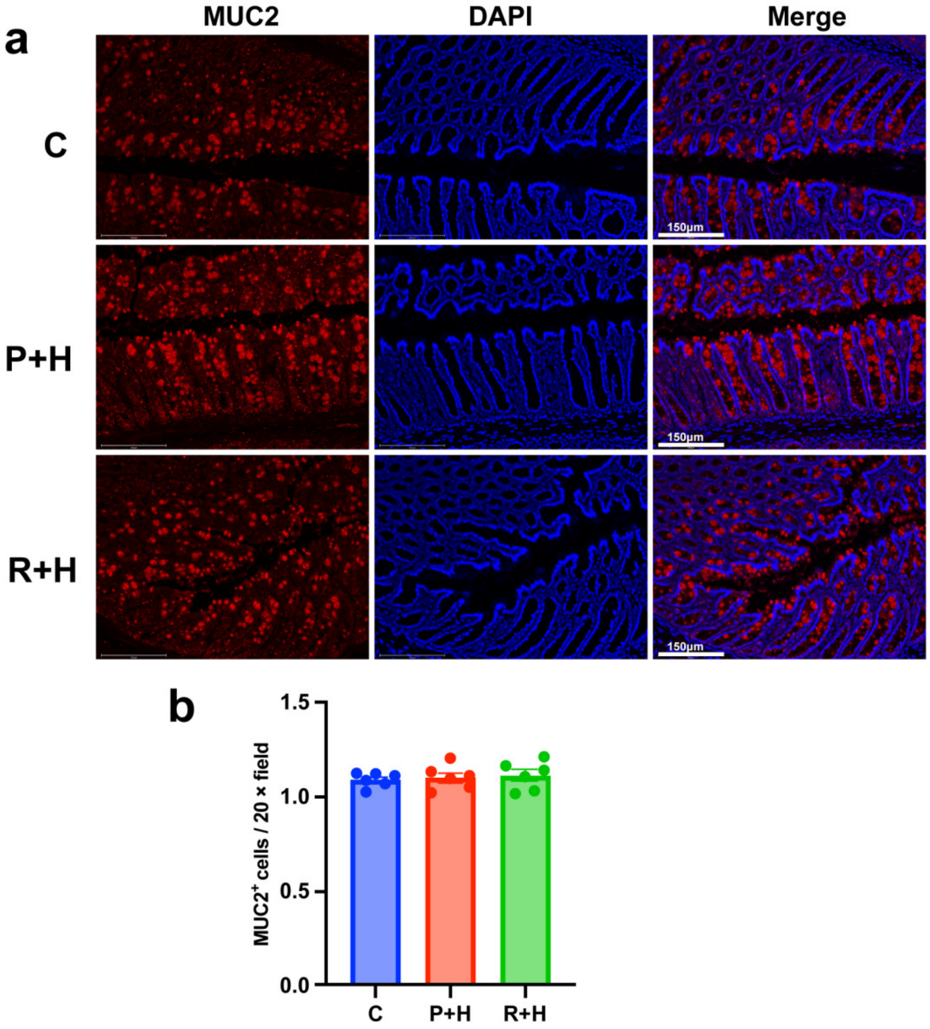

Figure S2 Colonic Histological Staining Results

AB/PAS-stained of mice in colon showing the goblet cells (a); Quantification of MUC2-positive cell intensities in the colon (b) was carried out using ImageJ software. The scale bar represents 100 μm in AB/PAS staining. Control group (C; n =6), PCPA group: P-H (150 mg/kg), n=6; Rosiglitazone group: R-H (30 mg/kg), n=6.

Table S1: Primers for real-time PCR

Gene name	Forword sequences (5'-3')	Reverse sequences (5'-3')
**M*uc2***	AGGGCTCGGAACTCCAGAAA	CCAGGGAATCGGTAGACATCG
**T*ff3***	TTGCTGGGTCCTCTGGGATAG	TACACTGCTCCGATGTGACAG
**K*lf3***	GAAGCCCAACAAATATGGGG	GGACGGGAACTTCAGAGAGG
**C*ryptdins***	GGTGATCATCAGACCCCAGCA	AAGAGACTAAAACTGAGGAGCAG
**D*efa6***	CCTTCCAGGTCCAGGCTGAT	TGAGAAGTGGTCATCAGGCAC
**L*yz1***	GCCAAGGTCTACAATCGTTGT	CAGTCAGCCAGCTTGACACCACG
**R*etnlb***	AAGCCTACACTGTGTTTCCTT	GCTTCCTTGATCCTTTGATCCAC
**N*lrp6***	TGACCAGAGCTTCCAGGAGT	TTTAGCAGGCCAAAGAGGAA
**C*aspase1***	CACAGCTCTGGAGATGGTGA	CTTTCAAGCTTGGGCACTTC
**A*sc***	ACAGAAGTGGACGGAGTGCT	CTCCAGGTCCATCACCAAGT
**A*tg5***	TGTGCTTCGAGATGTGTGGTT	ACCAACGTCAAATAGCTGACTC
**A*tg7***	TGACCTTCGCGGACCTAAAGA	CCCGGATTAGAGGGATGCTC
**A*tg12***	GAAGGCTGTAGGAGACACTC	GGAAGGGGCAAAGGACTGATTC
**B*eclin1***	ATGGAGGGGTCTAAGGCGTC	TCCTCTCCTGAGTTAGCCTCT
**P*62***	GAACTCGCTATAAGTGCAGTG	AGAGAAGCTATCAGAGAGGTGG
Gapdh	AGCTTGTCATCAACGGGAAG	TTTGATGTTAGTGGGGTCTCG

Supplementary Table S2. Antibodies used in this study.

Antibodies			
Target protein	Vendor	Catalog No.; RRID	WB working dilution
rabbit anti-PPAR-γ	Proteintech	Cat# 16643-1-Ap; RRID: AB_10596794	1:1000
mouse anti-β-catenin	Proteintech	Cat# 66379-1-Ig; RRID: AB_2857358	1:1000
Goat Anti-Rabbit Alexa fluor 594	Abcam	Cat# ab150080; RRID: AB_2650602	1:1000
Goat Anti-Mouse Alexa fluor 488	Abcam	Cat# ab150113; RRID: AB_2576208	1:1000
rabbit anti-MUC2	Abcam	Cat# ab272692; RRID: AB_2888616	1:1000
rabbit anti-Claudin-3	Abcam	Cat# ab15102; RRID: AB_301648	1:1000
rabbit anti-Claudin-1	Abcam	Cat# ab15098; RRID: AB_301644	1:1000
rabbit anti-Occludin	Abcam	Cat# ab216327; RRID: AB_2737295	1:1000
rabbit anti-TPH1	Abcam	Cat# ab52954; RRID: AB_2207555	1:1000
rabbit anti-SERT	Proteintech	Cat# 19559-1-AP; RRID: AB_2878590	1:1000
rabbit anti-PPAR-γ	Proteintech	Cat# 16643-1-AP; RRID: AB_10596794	1:1000
rabbit anti-NLRP6	Abcam	Cat# ab58705; RRID: AB_2152559	1:1000
rabbit anti-Caspase-1	Proteintech	Cat# 22915-1-AP; RRID: AB_2876874	1:1000
mouse anti-ATG16L	Proteintech	Cat# 67943-1-Ig; RRID: AB_2918695	1:1000
rabbit anti-LC3	Proteintech	Cat# 14600-1-AP; RRID: AB_2137737	1:1000
mouse anti-SQSTM1/p62	Santa	Cat# 88588; RRID: AB_2800125	1:1000
mouse anti-ATG7	Proteintech	Cat# 10088-2-AP; RRID: AB_2062351	1:1000
rabbit anti-ATG5	Proteintech	Cat# 10181-2-AP; RRID: AB_2062045	1:1000
mouse anti-β-actin	Proteintech	Cat# 66009-1-lg; RRID: AB_2687938	1:8000
goat anti-mouse IgG	Proteintech	Cat# SA00001-1; RRID: AB_2722565	1:10000
goat anti-rabbit IgG	Proteintech	Cat# SA00001-2; RRID: AB_2722564	1:10000

Fig1 f

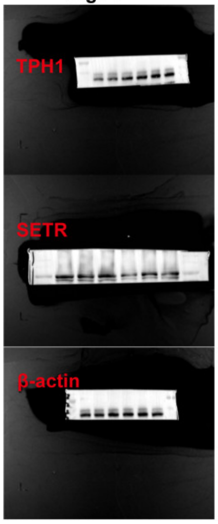

Fig1 g

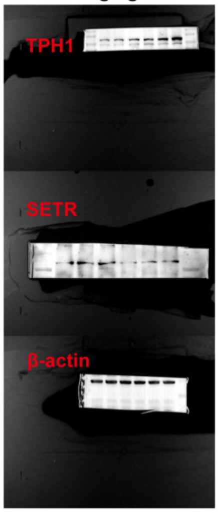

Fig2 e

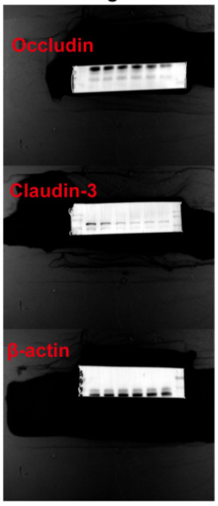

Fig3 d

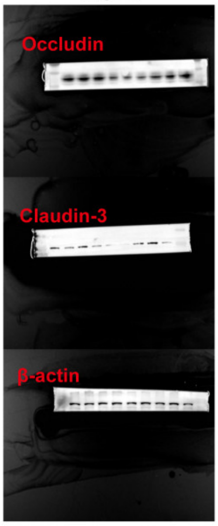

Fig4 c

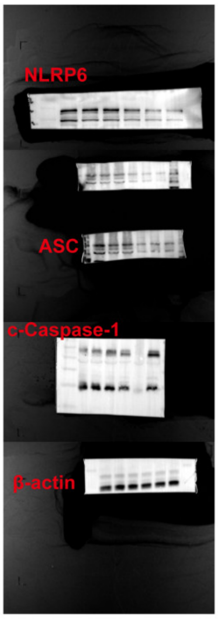

Fig5 c

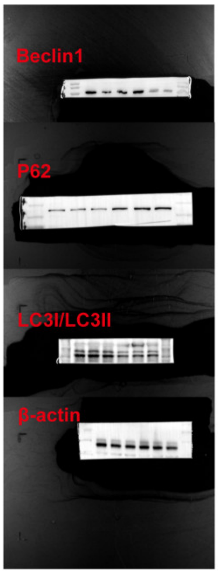

Fig6 e

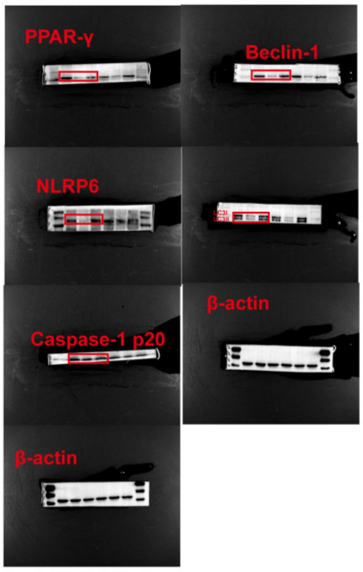

Fig6 g

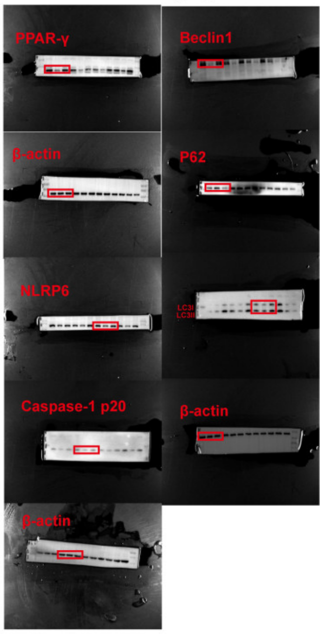

Fig7 a

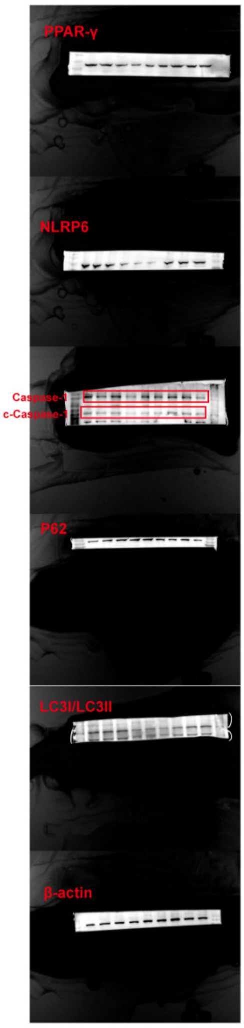

Fig7 g

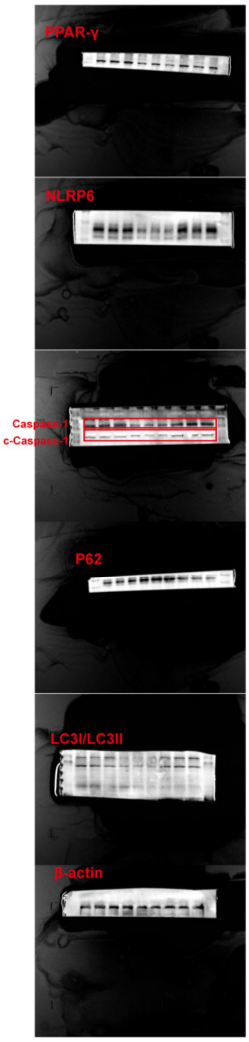

Fig9 c

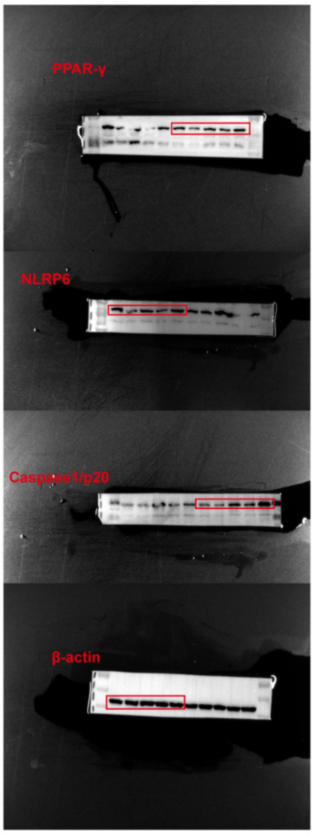

Fig10

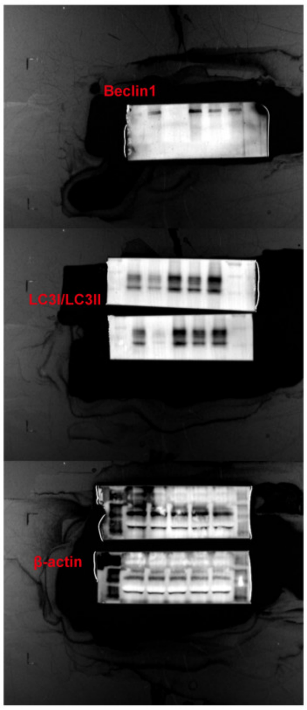

Figure S3 Original Western blot images for Figures 1–7, 9, 10
